# The research landscape of tuberous sclerosis complex–associated neuropsychiatric disorders (TAND)—a comprehensive scoping review

**DOI:** 10.1186/s11689-022-09423-3

**Published:** 2022-02-13

**Authors:** Stephanie Vanclooster, Stacey Bissell, Agnies M. van Eeghen, Nola Chambers, Liesbeth De Waele, Anna W. Byars, Jamie K. Capal, Sebastián Cukier, Peter Davis, Jennifer Flinn, Sugnet Gardner-Lubbe, Tanjala Gipson, Tosca-Marie Heunis, Dena Hook, J. Christopher Kingswood, Darcy A. Krueger, Aubrey J. Kumm, Mustafa Sahin, Eva Schoeters, Catherine Smith, Shoba Srivastava, Megumi Takei, Robert Waltereit, Anna C. Jansen, Petrus J. de Vries

**Affiliations:** 1 Department of Public Health, Mental Health and Wellbeing Research Group, Vrije Universiteit Brussel, Brussels, Belgium; 2 Cerebra Network for Neurodevelopmental Disorders, University of Birmingham, Birmingham, UK; 3 Emma Children’s Hospital, Amsterdam University Medical Center, Amsterdam, The Netherlands; 4 TAND Expert Centre, ‘s Heeren Loo, Hoofddorp, The Netherlands; 5 Division of Child & Adolescent Psychiatry, Centre for Autism Research in Africa (CARA), University of Cape Town, Cape Town, South Africa; 6 Department of Paediatric Neurology, University Hospitals Leuven, Leuven, Belgium; 7 Department of Development and Regeneration, KU Leuven, Leuven, Belgium; 8 Department of Pediatrics, Division of Neurology, Cincinnati Children’s Hospital Medical Center/University of Cincinnati College of Medicine, Cincinnati, OH USA; 9 Department of Neurology, University of North Carolina at Chapel Hill, Chapel Hill, NC USA; 10 Argentine Program for Children, Adolescents and Adults with Autism Spectrum Disorders (PANAACEA), Buenos Aires, Argentina; 11 Department of Neurology, Harvard Medical School & Boston Children’s Hospital, Boston, MA USA; 12 TSC Canada, Mississauga, Ontario Canada; 13 Stellenbosch University, Stellenbosch, South Africa; 14 Department of Pediatrics, University of Tennessee Health Sciences Center, Memphis, TN USA; 15 Le Bonheur Children’s Hospital and Boling Center for Developmental Disabilities, Memphis, TN USA; 16 TSC Alliance, Silver Spring, MD USA; 17 St. George’s University of London, London, UK; 18 The Royal Sussex County Hospital, Brighton, UK; 19 TSC Clinic Cincinnati Children’s Hospital Medical Center, Cincinnati, OH USA; 20 Department of Pediatrics, Clinical Pediatrics and Neurology, University of Cincinnati College of Medicine, Cincinnati, OH USA; 21 Department of Neurology, Translational Neuroscience Center, Boston Children’s Hospital, Harvard Medical School, Boston, MA USA; 22 Belgian TSC Association (Be-TSC), Mortsel, Belgium; 23 Tuberous Sclerosis Alliance India, Mumbai, India; 24 Japanese Society of Tuberous Sclerosis Complex, Tokyo, Japan; 25 Child and Adolescent Psychiatry, University Medical Center Göttingen, Georg-August University, Göttingen, Germany; 26 Department of Pediatrics, Pediatric Neurology Unit, Antwerp University Hospital, Edegem, Belgium

**Keywords:** Tuberous sclerosis complex, Scoping review, TSC-associated neuropsychiatric disorders, Autism, Behaviour, Psychiatric, Intellectual, Scholastic, Neuropsychological, Psychosocial

## Abstract

**Background:**

Tuberous sclerosis complex (TSC)–associated neuropsychiatric disorders (TAND) is an umbrella term for the behavioural, psychiatric, intellectual, academic, neuropsychological and psychosocial manifestations of TSC. Although TAND affects 90% of individuals with TSC during their lifetime, these manifestations are relatively under-assessed, under-treated and under-researched. We performed a comprehensive scoping review of all TAND research to date (a) to describe the existing TAND research landscape and (b) to identify knowledge gaps to guide future TAND research.

**Methods:**

The study was conducted in accordance with stages outlined within the Arksey and O’Malley scoping review framework. Ten research questions relating to study characteristics, research design and research content of TAND levels and clusters were examined.

**Results:**

Of the 2841 returned searches, 230 articles published between 1987 and 2020 were included (animal studies = 30, case studies = 47, cohort studies = 153), with more than half published since the term TAND was coined in 2012 (118/230; 51%). Cohort studies largely involved children and/or adolescents (63%) as opposed to older adults (16%). Studies were represented across 341 individual research sites from 45 countries, the majority from the USA (89/341; 26%) and the UK (50/341; 15%). Only 48 research sites (14%) were within low–middle income countries (LMICs). Animal studies and case studies were of relatively high/high quality, but cohort studies showed significant variability. Of the 153 cohort studies, only 16 (10%) included interventions. None of these were non-pharmacological, and only 13 employed remote methodologies (e.g. telephone interviews, online surveys). Of all TAND clusters, the autism spectrum disorder–like cluster was the most widely researched (138/230; 60%) and the scholastic cluster the least (53/200; 27%).

**Conclusions:**

Despite the recent increase in TAND research, studies that represent participants across the lifespan, LMIC research sites and non-pharmacological interventions were identified as future priorities. The quality of cohort studies requires improvement, to which the use of standardised direct behavioural assessments may contribute. In human studies, the academic level in particular warrants further investigation. Remote technologies could help to address many of the TAND knowledge gaps identified.

**Supplementary Information:**

The online version contains supplementary material available at 10.1186/s11689-022-09423-3.

## Background

Tuberous sclerosis complex (TSC) is an autosomal dominant genetic disorder characterised by multisystem involvement [1, 2]. The most common physical manifestations include benign tumours in the central nervous system, skin, kidneys, heart and lungs and high rates of epilepsy [2, 3]. TSC is caused by a pathogenic variant in one of two genes, *TSC1* or *TSC2* [4, 5]. The protein products of TSC form an intracellular complex to regulate mammalian/mechanistic target of rapamycin (mTOR) signalling [1, 6]. Dysregulation of mTOR signalling leads to overactivated mTOR as the core molecular mechanism of the disorder [7–9]. Among individuals with TSC, there is significant phenotypic variability in the number and severity of symptoms [3, 8]. Some physical characteristics of the disorder have an age-related expression pattern with cardiac rhabdomyomas, subependymal nodules and cortical tubers often emerging prenatally or in early infancy, and renal angiomyolipomas and lymphangioleiomyomatosis presenting more often in adolescence and adulthood [10, 11]. Evidence-based management and co-ordination of care across medical specialists is crucial throughout the lifespan to reduce morbidity and mortality in TSC [8, 12].

In addition to the physical manifestations of TSC, the disorder is also associated with a wide range of behavioural, psychiatric, intellectual, academic, neuropsychological and psychosocial difficulties [1, 13–15]. Collectively, these are referred to as TSC-associated neuropsychiatric disorders or ‘TAND’, a term coined in 2012 [13]. Approximately 90% of people with TSC evidence TAND manifestations at some point in their lives, and TAND has been identified by families as the greatest clinical burden of the disorder [13, 16–18]. Similar to the physical manifestations, TAND also has an age-related expression with some difficulties or disorders more prevalent in infancy or early childhood (e.g. impulsivity and overactivity), and others emerging or presenting later across the lifespan (e.g. anxiety and depressed mood) [13–16, 19]. Although a genotype–intellectual phenotype relationship is well delineated in TSC, with a more severe phenotype associated with the *TSC2* variant, a *TSC1 TSC2* differentiation is overly simplistic in relation to TAND. There is a complex multi-directional association between the physical and neuropsychiatric aspects of TAND. As discussed in more detail below, seizure severity, intellectual ability, developmental outcomes and autism characteristics are interrelated [20]. There is also a humanistic impact to educational, social, psychological and quality of life outcomes as a result of physical health determinants, including epilepsy, medication side effects and pain [21].

### A brief history of TAND research

From a historical perspective, the association between TSC and TAND has been noted from very early on in the narrative of TSC research. On the first description of ‘sclérose tubéreuse des circonvolutions cérébrales’ (tuberous sclerosis of the cerebral cortex), TSC was conceptualised as a disorder of the brain [22]. Only when Vogt [23] described the ‘triad of impairment’ (seizures/epilepsy, intellectual disability and angiofibromas of the skin) did TSC become a disorder of multisystem involvement. Early descriptive studies by Sherlock [24] and Critchley and Earl [25] in particular, showed clear associations between TSC and a range of behaviours that would now be associated with psychopathologies such as autism, attention deficit hyperactivity disorder (ADHD) or psychotic disorders. Early diagnostic criteria, however, only described the physical manifestations of TSC [26]. The pioneer of systematic research on the behavioural aspects of TSC was the UK scientist, parent and co-founder of the UK Tuberous Sclerosis Association (TSA), Ann Hunt, who in 1983 published a set of papers on behaviours and family perspectives [27–29], and later published research exploring behavioural ‘risk markers’ [30, 31]. Jambaqué et al. in France [32] described the first systematic evaluation of neuropsychological profiles in TSC and showed that over and above intellectual disability, many people with TSC (including those with normal intellectual ability) had specific neuropsychological deficits in memory, attention, language and executive skills.

The link between physical and neuropsychiatric aspects of TSC became more established with improvements in neuroimaging techniques. Research in the mid–late 1990s explored correlations between structural brain abnormalities such as cortical tubers and a range of TAND-related manifestations, most notably autism and intellectual disability (e.g. [33, 34]). The first set of consensus diagnostic criteria for TSC in 1998 [35, 36] acknowledged the presence of neurodevelopmental concerns and recommended ‘thorough age-appropriate screening for behavioural and neurodevelopmental dysfunction’ at diagnosis and re-evaluation ‘as indicated’. TAND research in the 2000s continued to describe the range of TAND manifestations and started to stratify and correlate these in relation to intellectual ability level, and/or seizures (e.g. [37, 38]). As research broadened across age groups, different manifestations of behaviour in TSC were found to be linked to different stages across the lifespan. This lifespan perspective and advocacy from parent organisations such as the TSA for proactive assessment of TAND, led to the generation of consensus clinical guidelines for the assessment of behavioural and cognitive problems in TSC [39]. These guidelines recommended comprehensive evaluation at key developmental timepoints (e.g. infancy, pre-school, primary years, adolescence, early adulthood) and urgent assessment in response to sudden and unexpected changes in behaviour or cognition. Later in that decade, specific profiles of behaviour were more extensively explored along with potential ‘risk markers’ for such behaviours [40–42].

After the identification of the role of the *TSC1* and *TSC2* genes in intracellular signalling in the mTOR signalling pathway [43, 44], TAND research started to shift focus to the potential role of molecular pathways to psychopathology. For example, de Vries and Howe published the ‘global regulator and integrator of a range of physiological processes’ (GRIPP) hypothesis [45] stating that structural and electrophysiological features of TSC are neither necessary nor sufficient to explain TAND manifestations and proposed that mTOR dysregulation may represent a direct pathway to TAND. Therefore, TAND manifestations may be reversed or improved by mTOR inhibitors (mTORi) or other molecularly targeted treatments [16, 45]. TAND research since then has examined animal models in relation to mTORi, and phase I and II clinical trials of mTORi in humans emerged [46]. In spite of initial encouraging findings of improvement in specific TAND manifestations in animal models and early-phase human trials [47–49], more recent results have been mixed [50, 51].

At the International TSC Consensus Conference in 2012, the Neuropsychiatry Panel recognised that the 2005 guidelines [39] were rarely followed and that the majority of neuropsychiatric manifestations in TSC were not identified or treated [8, 13].

Here, the emphasis on psychosocial aspects of TSC and ‘burden of illness’ gained precedence. The financial, humanistic, and quality of life impacts to individuals, caregivers and families were noted, including healthcare costs, caregiver stress and school absenteeism [52, 53]. Individuals and caregivers reported stressors in relation to both physical characteristics (e.g. skin lesions, tumour burden, epilepsy) and TAND (e.g. poor sleep, stigma, depression, social isolation [54]). Physical characteristics such as active seizure status, adverse medication side effects, and TSC-specific severity of manifestations have been found to predict health-related quality of life and TAND outcomes (e.g. social involvement, emotional well-being, cognitive functioning [55]). Caregivers in particular noted concerns regarding transition into adult services and a lack of multidisciplinary involvement and specialist care [54].

There was therefore a clear ‘identification and treatment gap’ of these manifestations [14]. In addition, the panel noted that there was significant confusion in the international literature about the many different ‘levels’ of neuropsychiatric manifestations, with differing terminology used across the globe. As a result, the panel coined the term ‘TAND’ for two reasons: firstly, to create a simple ‘umbrella’ term to capture the wide range of neuropsychiatric manifestations associated with TSC and, secondly, to provide a ‘shared language’ to define the different levels of TAND [13]. Consequently, TAND was included as a core component of an international patient registry [19, 56, 57] and was outlined as a recommendation for future research as of 2016 [58]. This timeline of TAND research to date is summarised in Table 1.

**Table 1:** Historical developments in TAND research

**1880**	TSC defined as a disorder of the brain [19]
**1908**	Description of the ‘triad of impairment’ which included seizures/epilepsy, intellectual disability and facial angiofibromas [20]
**1911**	The term ‘epiloia’ coined to describe epilepsy combined with ‘anoia’ (intellectual disability) in individuals with TSC [21]
**1932**	First descriptions of behaviours suggestive of autism, behavioural manifestations and different intellectual levels in individuals with TSC [22]
**1967**	First set of diagnostic criteria for TSC—not including any TAND manifestations or reference to seizures/epilepsy [23]
**1983**	First systematic research on behavioural aspects of TSC [24–26]
**1987**	Exploration of infantile spasms and its relationship with behavioural manifestations in TSC (e.g. autism, hyperkinetic behaviour, psychosis and aggression) [27]
**1991**	Consideration of neuropsychological deficits in TSC, in relation to memory, attention and executive functions [29]
**1993**	Further exploration of links between TSC and varied behavioural problems and identification of risk markers of behavioural manifestations [28]
**1998**	First International TSC Consensus Conference to develop revised diagnostic criteria and clinical management guidelines with little consideration of TAND [32, 33]
**2005**	TSC Behaviour Consensus Panel publish clinical guidelines for the assessment of cognitive and behavioural problems in TSC: recommendations of comprehensive assessment during all key developmental phases to identify emerging TAND and urgent assessment in case of sudden or unexpected change [36]
**2007**	Molecular hypothesis for the causes of TAND: the GRIPP hypothesis proposed that there is a direct molecular pathway from gene disruption to psychopathologies and that molecularly targeted treatments may reverse these deficits [42]
**2008**	First animal models of *TSC2+/-* showing reversal of learning deficits in response to mTORi [45]
**2011**	First human findings to show improvement in memory and executive deficits in humans with TSC after mTORi in an open-label trial [44]
**2012**	Second International TSC Consensus Conference to revise diagnostic criteria, as well as surveillance and treatment guidelines for TSC [8] The term ‘TAND’ was coined, and the recommendation was made to screen for TAND on an annual basis [9].
**2012**	Establishment of the TuberOus SClerosis registry to increase Awareness (TOSCA) consortium: the first large-scale international collaboration to study physical and TAND manifestations [49–51]
**2015**	Pilot validation and publication of the TAND Checklist [14, 17] with subsequent translation and authorisation in 19 languages (http://www.tandconsortium.org)
**2016**	Inclusion of TAND in Research Strategic Plan for TSC [52]
**2017**	First publication of randomised controlled trial findings on TAND from everolimus and sirolimus clinical trials [47]
**2018**	First description of natural TAND clusters [53]
**2019**	Launch of the TANDem project and establishment of the TAND consortium (http://www.tandconsortium.org)
**2020**	Replication of natural TAND clusters [54]
**2021**	Updated TSC Diagnostic Criteria and Surveillance and Management Recommendations including consensus guidelines for the identification and treatment of TAND [12]

*GRIPP* Global regulator and integrator of a range of physiological processes, *mTORi* Mechanistic target of rapamycin inhibitors, *TANDem* ‘Empowering families through technology: a mobile-health project to reduce the TAND identification and treatment gap’

Table 2 presents a summary of the different levels of TAND as defined by the Neuropsychiatry Panel [8]. Given the possible changes over time in an individual’s TAND profile, it was recommended that all individuals with TSC should be screened for TAND at least annually [8]. The TAND Checklist was developed to guide healthcare practitioners in screening across the different levels of neuropsychiatric functioning [13, 17].

**Table 2:** The different levels of TAND

Level	Name	Description	Examples
**Level 1**	Behavioural level	This level includes all observed behaviours. The behavioural level is typically evaluated through direct observation or through a range of rating scale measures.	Aggression, anxiety, depressed mood, overactivity, impulsivity, poor eye contact, repetitive and ritualistic behaviours, sleep problems
**Level 2**	Psychiatric level	This level is defined by psychiatric diagnostic classification systems such as DSM-5 or ICD-11. At this level, the clinician determines whether behaviours observed at level 1 meet criteria for specific psychiatric disorders.	ADHD, autism, anxiety disorder, depressive disorder
**Level 3**	Intellectual level	This level measures intellectual ability as defined by standardised IQ-type measures.	Intellectual ability within the normal, mild, moderate, severe or profound range.
**Level 4**	Academic level	This level refers to specific learning disorders (as defined in DSM-5) associated with scholastic performance.	Reading, writing, spelling, or mathematics disorder.
**Level 5**	Neuropsychological level	This level examines specific brain-referenced systems through the use of standardised neuropsychological instruments.	Selective, sustained or dual-tasking attention deficits; unilateral neglect; immediate recall memory deficits; spatial working memory deficits; visuo-spatial deficits; executive deficits
**Level 6**	Psychosocial level	This level explores the psychological and social impact of TSC in terms of self, family and community relationships.	Low self-esteem, low self-efficacy, high family stress, parental relationship difficulties, community stigma and isolation

*DSM-5* Diagnostic and Statistical Manual of Mental Disorders, Fifth Edition [59], *ICD-11* International Classification of Diseases and Related Health Problems, Eleventh Edition [60]

Apart from the ‘assessment and treatment gap’ described in TAND, it was also clear that the wide range of TAND manifestations across the lifespan presented an almost ‘overwhelming uniqueness’ of TAND profiles resulting in a ‘treatment paralysis’, as described by Leclezio and de Vries [14]. The authors used a data-driven strategy to identify natural clusters of TAND manifestations with a view towards creating a smaller number of typical TAND profiles that could guide identification and intervention for TAND [61]. Following feasibility [61] and replication [62], findings from a large-scale study of 453 participants revealed seven natural TAND clusters: a scholastic cluster, a neuropsychological cluster, a dysregulated behaviour cluster, an overactive/impulsive cluster, an eat/sleep cluster, a mood/anxiety cluster and an autism spectrum disorder–like cluster [63]. The clusters represent typically occurring groupings of the specific manifestations associated with the six different levels of TAND, as shown in Table 2. Table 3 outlines these seven natural TAND clusters and their items.

**Table 3:** The seven natural TAND clusters and their items

TAND clusters	TAND items
1. Scholastic	Reading, writing, spelling, mathematics
2. Neuropsychological	Memory, disorientation, attention deficits (behavioural and neuropsychological), visuo-spatial deficits, dual-task deficits, executive function deficits
3. Dysregulated behaviour	Aggressive outbursts, temper tantrums, self-injury
4. Overactive/impulsive	Overactivity, impulsivity, restlessness
5. Eat/sleep	Eating difficulties, sleep difficulties
6. Mood/anxiety	Anxiety, depressed mood, extreme shyness, mood swings
7. Autism spectrum disorder–like	Inflexibility, unusual language, delayed language, repetitive behaviours, poor eye contact, peer difficulties

It is important to acknowledge the conceptual distinction between TAND levels and clusters. TAND levels distinguish between six discrete aspects relating to the neuropsychiatric characteristics of TSC. This is a simple and heterogeneous approach, which is pragmatic within a clinical context to ensure each level is explored within the TAND Checklist. Levels ‘categorise’ discrete TAND manifestations, but it is important to note that each level does not occur in isolation. Clusters by comparison consider the overlap and co-occurrence of items beyond levels, in reference to an individual’s TAND profile or ‘signature’. Clusters ‘group’ naturally co-occurring TAND manifestations based on large-scale data modelling [63], with a view towards more personalised identification and treatment.

### Describing the research landscape of TAND

The last three decades have seen progress in the field of TAND research. However, as previously outlined, TAND is a broad and complex construct. Despite the increased interest and output in TAND, there has been no comprehensive synthesis of TAND findings to guide clinical decision making or directions for future research. Table 4 outlines the key unanswered ‘big picture’ questions in the field. These research questions are presented in chronological order of their relative impact to the TSC research field in line with the background history of TAND research. Both TAND levels and clusters are clinically relevant; however, TAND levels are comparatively more established in accordance with TAND Checklist structure and content [13]. Exploring TAND levels within a scoping review context also enables a consideration of important psychosocial aspects of TSC that are not fully encapsulated within the TAND cluster framework. Therefore, research questions are mainly addressed in relation to TAND levels, with a brief exploration of clusters in the last question. The current review set out to identify and evaluate all TAND research to date, with the aims of: (a) describing the research landscape of TAND and (b) finding knowledge gaps in TAND research that could inform priority setting and recommendations for future TAND research.

**Table 4:** Key scoping review questions

1. How much TAND research has been done over the years?	
2. Where has TAND research been done in the world?	
3. Which TSC age groups have been investigated?	
4. What is the overall quality of existing TAND research?	
5. Which TAND levels have been investigated?	
6. Which research methods and research measures have been used to investigate TAND?	
7. How much quantitative and qualitative TAND research has been conducted?	
8. How many intervention studies have been conducted?	
9. Have remote technologies been utilised to study TAND?	
10. Which TAND clusters have been studied?	

## Methods

### Scoping review methodology

To investigate these two key aims and directly address the ten research questions outlined in Table 4, a scoping review methodology was adopted. A key component of a scoping review is ‘mapping’ [64]: synthesising what is currently known about a research topic, identifying gaps in current understanding and conceptually analysing the available literature to inform the focus of future research [65, 66]. Unlike systematic reviews, studies in a scoping review are not excluded on the basis of quality, although it is generally recommended that methodological quality of studies should be assessed to aid in the interpretation of scoping review results [64]. The five stages of the Arksey and O’Malley framework [67] for conducting a scoping review were followed (outlining research questions, study identification, study selection, data charting, analysis and interpretation). In addition, a quality assessment of studies was also conducted.

### Study identification

A systematic search of the following databases was conducted by the co-first author (SV) between February and March 2020: CINAHL, Cochrane Library, Embase, ERIC, MEDLINE, PsycARTICLES, PubMed, Sage Journals, Science Direct, Scopus (Elsevier), Springer Link and Web of Science. Searches were conducted using TSC search term variations and keywords encompassing each of the six levels of TAND. TSC and TAND level search terms were combined with the Boolean operator ‘AND’, as outlined in Additional file 1.

### Study selection

The titles and abstracts of the returned searches were screened for relevance according to the following inclusion criteria: (1) scientific articles published in peer-reviewed scientific journals (including case reports, letters to editors, research letters and research commentaries), (2) full-text access available, (3) TAND level keywords included in the resource subject, (4) any research methodology or study design containing primary TAND data (including theoretical and applied basic research, descriptive studies, interventions, qualitative, quantitative and mixed method approaches) and (5) relevant TSC study populations described (including animal models, individuals with TSC, families/caregivers and healthcare providers). No language or date limitations were imposed during initial screening or full-text review. Exclusions were made if search returns were: (1) related to non-TAND topics (e.g. genetic mechanisms of TSC), (2) literature reviews, systematic reviews or meta-analyses with no reference to primary TAND data, or (3) obtained from grey literature resources with no reference to primary TAND data (e.g. commentaries, reports, book chapters or conference proceedings). Grey literature is defined as work that is not formally published under the control of commercial organisations (e.g. academic journals [68]). Although there is increasing focus on the value of including grey literature in systematic reviews and meta-analyses to overcome publication bias [69], there is less of an emphasis on its inclusion in scoping reviews when synthesising what is broadly known about a research topic. Only half of scoping reviews currently include grey literature [70], with the allocation of time and resources conserved for subsequent comprehensive systematic reviews [71].

**Fig. 1:**
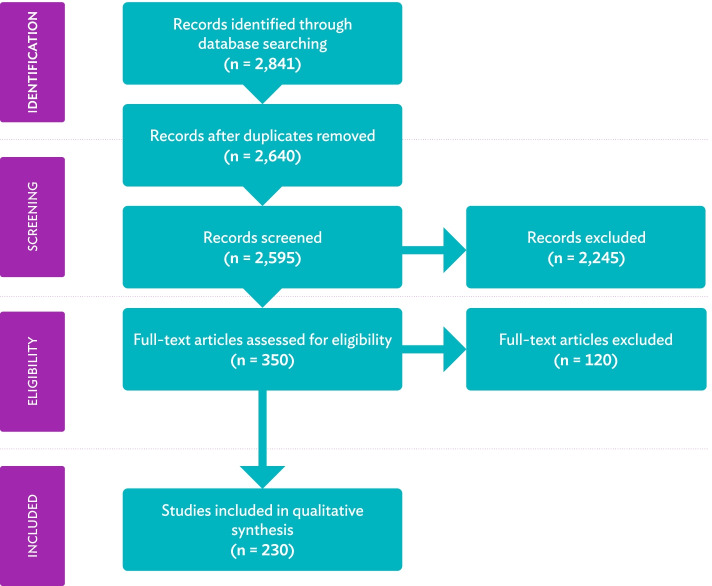
PRISMA flow diagram of study selection. Please note that articles are referred to as ‘records’ before full-text screening and as ‘studies’ once included in the review

Based on this initial screening, 2245 search returns were excluded, as titles did not reference TAND level keywords. Following the removal of duplicates (*n* = 201) and search returns from reviews and grey literature sources (*n* = 45), 350 records were considered for full-text review. The inclusion and exclusion criteria outlined above were also applied during the full-text review. The two co-first authors (SV and SB) independently completed the full-text review process to verify the accuracy of the screening procedure. Where eligibility of a study was unclear, inclusion/exclusion criteria were discussed between the main authors to reach consensus. When consensus could not be reached (*n* = 9), inclusion or exclusion was determined by the senior authors (AJ and PdV). This resulted in the inclusion of 230 eligible studies in this scoping review (see Fig. 1). Full references of all 230 included studies are provided in Additional file 2.

### Data charting

A table for data extraction was developed at the full-text review stage, with study characteristics identified in accordance with the ten research questions outlined. Given substantial differences in data that can be extracted from animal studies, case studies (descriptive non-statistical clinical observations) and cohort studies (analytical observational or experimental designs with single or multiple groups), three distinct data extraction tables were utilised to allow for such differences in study type. Studies were therefore distinguished according to animal studies (30/230), case studies (47/230) and cohort studies (153/230). Here, a distinction is made between case and cohort studies, whereby case studies are in-depth systematic evaluations of a single person or group without the presentation of statistical data analyses, and cohort studies involve groups of individuals with TSC taking part in experimental, non-experimental, observational, follow-up and case review study designs where group-level statistical data are presented. Data extraction was performed by the co-first authors (50% SV and 50% SB). It is important to note that studies could span multiple TAND levels and clusters (e.g. autism profile studies that also referenced behavioural dysregulation). For this reason, results reported here can exceed the maximum number of studies per study type. For example, there were 153 cohort studies in total and 93 reference behavioural TAND level information and 71 reference psychiatric TAND level information. Some of these studies would have referenced both behavioural and psychiatric information. Results are therefore not summative but reported as percentages of the total number of cohort studies (93/153; 61% and 71/153; 46%), which can exceed 153 (100%). In relation to research question eight, behaviours reported as a consequence of an intervention (e.g. fatigue and vomiting as adverse events of everolimus) were not extracted as primary TAND data. However, baseline pre-intervention behaviours (e.g. aggression, self-injury) were extracted when reported.

### Quality appraisal

To address research question four, the quality of all 230 included studies was evaluated. Animal studies were appraised using the Animal Research: Reporting of In Vivo Experiments 2.0 guidelines (ARRIVE) [72], and case studies and cohort studies were appraised using the Mixed Methods Appraisal Tool (MMAT) [73]. When utilising such tools, quantitatively rating each criterion to establish an overall quality score is discouraged. Instead, authors (SV, SB, NC, AVE) provided qualitative information for each criterion, and studies were grouped according to quality based on these descriptive summaries. For further information regarding the grouping of studies according to quality, please refer to Additional file 3.

### Inter-rater reliability

Inter-rater reliability was established at study selection, data charting and quality appraisal stages according to Cohen’s kappa coefficient (κ), where *κ* = (*p*_*o* –_*p*_*e*_) / 1 – *p*_*e*_). Inter-rater reliability is considered good at ≥ .61 and excellent at ≥ .81. Inter-rater reliability of full-text screening between SV and SB was good (*κ* = .709). Inter-rater reliability of data extraction content for TAND levels and clusters was calculated for over half of the included studies (128/230; 56%) to establish whether the same information was reliably inferred. Overall level of agreement between the co-first authors (SV and SB) was excellent (animal studies: *κ* = 1.000, case studies: *κ* = .827, cohort studies: *κ* = .839). Where discrepancies in data extraction occurred, differences were discussed and adjusted by consensus. Thirty cohort studies, 10 case studies and 6 animal studies (46/230; 20% of all included studies) were assessed for quality by the co-first author (SB) to establish quality appraisal inter-rater reliability with the primary raters (SV, NC, AVE). Inter-rater reliability was excellent for case studies (*κ* = .857), fair for cohort studies (*κ* = .277) and moderate for animal studies (*κ* = .423).

## Results

### Research question 1: how much TAND research has been done over the years?

**Fig. 2:**
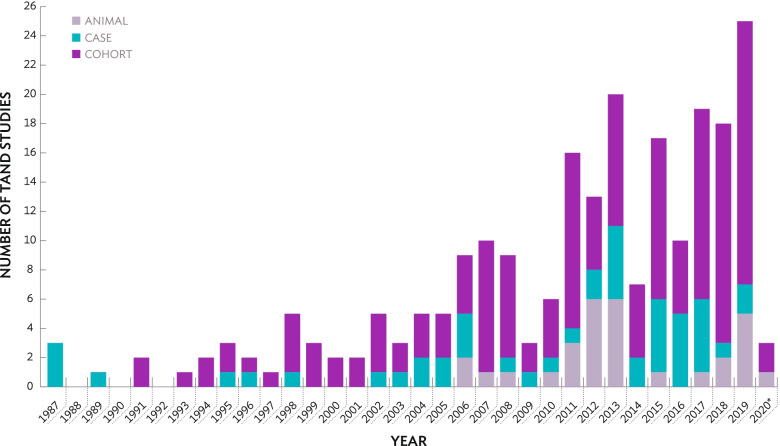
Number of TAND studies across years based on study type. *The search was completed in March 2020. As a result, the column does not represent all TAND studies published in 2020

A total of 230 articles were identified that met the inclusion criteria for the review. As shown in Fig. 2, three case reports were published in 1987 describing intellectual ability and autism profiles [74–76]. In 1991, two UK cohort studies describing the behavioural, intellectual and academic levels of TAND were published by Webb; the first paper profiling intellectual ability and autism in TSC [77] and the second utilising magnetic resonance imaging (MRI) to explore the neuroimaging profile of children and adults of ‘normal intellect’ [78]. It is important to note, several earlier TAND papers outlined in Table 1 (e.g. [25, 29]) were not identified during the search. The first animal studies of TAND were published in 2006 [79, 80]; both explored memory processing in the Eker rat, a naturally occurring *Tsc2+/-* rat model. Taking together animal, case and cohort studies, Fig. 2 shows a clear increase in TAND research, which is particularly pronounced from 2013 onwards (118/230; 51%) after the term TAND was coined in 2012.

### Research question 2: where has TAND research been done in the world?

**Fig. 3:**
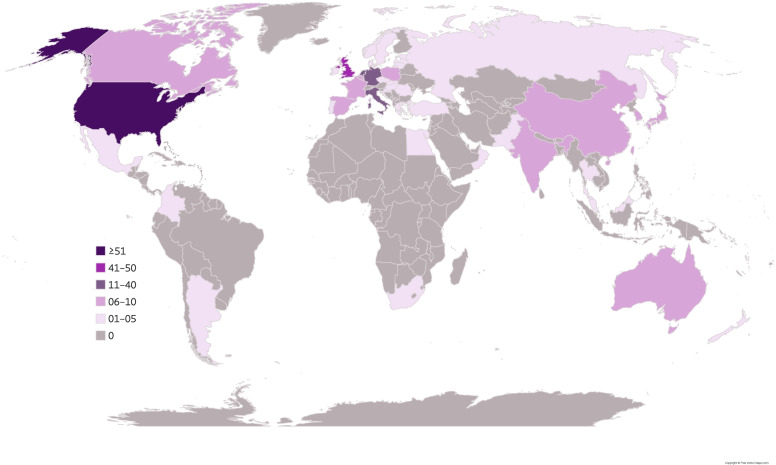
World map depicting the geographical location of the 341 research sites identified across the 230 studies. Darker colours depict a greater number of research sites per country

Countries were categorised as either a high-income country (HIC) or low–middle-income country (LMIC) according to the World Bank List of Economies [81]. Here, geographical location refers to the countries where participants were recruited from, as opposed to the nationality or academic institution of listed authors. Geographical information was derived from the methods section of each study, as opposed to listed author information that may have included international co-author collaboration. International multisite studies include multiple research sites as participants were actively recruited from several countries; therefore, the number of research sites exceeds the total number of included studies. The majority of research, regardless of study type, came from HIC research sites (see Table 5). Efforts to include LMICs in TAND research has mainly been led by five international multisite studies in recent years [11, 19, 57, 61, 82]. Samples derived from community-based approaches largely involved European and American cohorts (e.g. Stichting Tubereuse Sclerosis Nederland, TSA UK, TSC Alliance USA). Only nine studies used a general population sample [34, 37, 83–89]. Of these, all were population-based studies from HICs (Italy, Sweden, UK, and USA). The geographical locations of all 230 studies included in the scoping review (represented as 341 individual research sites) are presented in Fig. 3 and Table 6.

**Table 5:** Study information of scoping review TAND s according to study type

	Animal studies (***n*** = 30)	Case studies (***n*** = 47)	Cohort studies (***n*** = 153)
Sample characteristics	*Species:* • Mice (*n* = 26; 87%) • Rats (*n* = 4; 13%)	*Sex:* • Male (*n* = 25; 53%) • Female (*n* = 14; 30%) • Multiple case series (*n* = 8; 17%) *Age distribution*^a^*:* • Infant 0–3 years (*n* = 9; 19%) • Child 4–10 years (*n* = 18; 38%) • Adolescent 11–19 years (*n* = 16; 34%) • Adult 20–60 years (*n* = 17; 36%) • Older adult 60+ years (*n* = 1; 2%)	*Sex ratio reported:* • 130 (85%) *Age distribution*^a^*:* • Infant 0–3 years (*n* = 90; 59%) • Child 4–10 years (*n* = 97; 63%) • Adolescent 11–19 years (*n* = 96; 63%) • Adult 20–60 years (*n* = 70; 46%) • Older adult 60+ years (*n* = 24; 16%) *Sample size:* • ≤ 50 (*n* = 88; 58%) • 51–100 (*n* = 28; 18%) • 101–200 (*n* = 17; 11%) • 201–500 (*n* = 13; 9%) • 501–1000 (*n* = 4; 3%) • ≥ 1001 (*n* = 3; 2%) *Mixed caregiver patient cohort:* • 7 (5%)
Clinical information	*Genetic information:* • TSC1 (*n* = 8; 27%) • TSC2 (*n* = 22; 73%)	*Epilepsy information provided:* • 39 (83%) *IQ information provided:* • 31 (66%) *Genetic confirmation provided:* • 11 (23%)	*Epilepsy information provided:* • All (*n* = 110; 72%) • Some (*n* = 14; 9%) • None (*n* = 29; 19%) *IQ information provided:* • All (*n* = 93; 61%) • Some (*n* = 21; 14%) • None (*n* = 39; 26%) *Genetic confirmation provided:* • All (*n* = 26; 17%) • Some (*n* = 34; 22%) • None (*n* = 93; 61%)
World Bank Classification	HIC (*n* = 30; 100%) LMIC (*n* = 0; 0%) Multisite HIC and LMIC N/A	HIC (*n* = 33; 70%) LMIC (*n* = 14; 30%) Multisite HIC and LMIC N/A	HIC (*n* = 138; 90%) LMIC (*n* = 10; 7%) Multisite HIC and LMIC (*n* = 5; 3%)
Sample identification^a^	N/A	N/A	Population (*n* = 9; 6%) Clinical (*n* = 108; 71%) Community (*n* = 47; 31%)
Quality rating	Relatively high (*n* = 8; 27%) High (*n* = 22; 73%)	Relatively high (*n* = 16; 34%) High (*n* = 31; 66%)	Low (*n* = 4; 3%) Adequate (*n* = 26; 17%) Relatively high (*n* = 84; 55%) High (*n* = 39; 25%)

^a^Studies could span multiple data extraction points (e.g. a study involving infants, children and adolescents); therefore, numbers and percentages reported within each category can exceed the maximum number of studies per study type. Percentages reported as percentage of study type total (animal studies: *n* = 30, case studies: *n* = 47, cohort studies: *n* = 153). *N/A* information not applicable to study type

**Table 6:** Number of TAND studies according to country (and study type)

Countries	Total (***A, CS, CO***)	Countries	Total (***A, CS, CO***)	Countries	Total (***A, CS, CO***)	Countries	Total (***A, CS, CO***)	Countries (S-U)	Total (***A, CS, CO***)
*Argentina*	1 (0, 0, 1)	*Denmark*	4 (0, 0, 4)	*Israel*	3 (0, 0, 3)	*Norway*	4 (0, 0, 4)	*South Africa*	5 (0, 0, 5)
*Australia*	9 (2, 0, 7)	*Egypt*	1 (0, 0, 1)	*Italy*	18 (1, 1, 16)	*Oman*	2 (0, 2, 0)	*South Korea*	8 (0, 2, 6)
*Austria*	4 (0, 0, 4)	*Estonia*	3 (0, 0, 3)	*Japan*	10 (1, 3, 6)	*Pakistan*	1 (0, 1, 0)	*Spain*	6 (0, 0, 6)
*Belgium*	6 (0, 0, 6)	*France*	9 (0, 2, 7)	*Latvia*	3 (0, 0, 3)	*Poland*	10 (0, 1, 9)	*Sweden*	5 (0, 0, 5)
*Canada*	7 (2, 0, 5)	*Germany*	14 (6, 0, 8)	*Lithuania*	3 (0, 0, 3)	*Portugal*	4 (0, 0, 4)	*Taiwan*	6 (0, 0, 6)
*China*	10 (0, 1, 9)	*Greece*	4 (0, 0, 4)	*Malaysia*	1 (0, 0, 1)	*Romania*	3 (0, 0, 3)	*Thailand*	4 (0, 0, 4)
*Colombia*	1 (0, 0, 1)	*Hungary*	1 (0, 0, 1)	*Mexico*	1 (0, 0, 1)	*Russia*	4 (0, 0, 4)	*Turkey*	4 (0, 0, 4)
*Croatia*	1 (0, 1, 0)	*India*	9 (0, 9, 0)	*Netherlands*	14 (0, 1, 13)	*Slovakia*	3 (0, 0, 3)	*UK*	50 (0, 5, 45)
*Czech Republic*	5 (0, 0, 5)	*Ireland*	1 (0, 0, 1)	*New Zealand*	1 (0, 1, 0)	*Slovenia*	3 (0, 0, 3)	*USA*	89 (21, 12, 56)

Countries listed in alphabetical order. *A* animal studies, *CS* case studies, *CO* cohort studies. Three hundred forty-one individual research sites across 230 studies depicted according to country, as multisite large-scale registry studies are also represented (e.g. Long-term, Prospective Study Evaluating Clinical and Molecular Biomarkers of Epileptogenesis in a Genetic Model of Epilepsy—Tuberous Sclerosis Complex (EPISTOP) and TuberOus SClerosis registry to increase disease Awareness (TOSCA)). Please note that specific European countries described in one study as ‘other European countries’ are not represented [90]

Overall, 341 individual research sites across 45 countries conducted TAND research. However, 41% of all TAND research was derived from just two countries: the USA (89/341; 26%) and the UK (50/341; 15%). Of note, no TSC animal studies came from the UK. A significant proportion of cohort participant research sites were located in Germany, Italy and the Netherlands. Countries defined as LMICs (Argentina, China, Colombia, Croatia, Egypt, India, Malaysia, Mexico, Pakistan, Romania, Russia, South Africa, Taiwan and Turkey) represented only a small combined proportion of research site involvement in published TAND research (48/341; 14%). Most LMICs were represented predominantly by international multisite studies as previously mentioned, as well as single-participant clinical case reports (e.g. [91]).

### Research question 3: which TSC age groups have been investigated?

As shown in Table 5, the majority of human TAND research involved school-age children aged 4–10 years and adolescent participant samples aged 11–19 years across case studies (school-age children: 18/47; 38%, adolescents: 16/47; 34%) and cohort studies (school-age children: 97/153; 63%, adolescents: 96/153; 63%). In contrast, very few case studies described or reported TAND manifestations in infants aged 0–3 years (9/47; 19%) and few case studies (1/47; 2%; [74]) or cohort studies (24/153; 16%) involving older adults over the age of 60 years.

### Research question 4: what is the overall quality of existing TAND research?

Based on the ARRIVE quality criteria, the majority of animal studies were rated as relatively high (8/30; 27%) or high quality (22/30; 73%). As outlined in Table 5, most case studies and cohort studies provided epilepsy and intellectual ability information of their participants; however, the number of individuals who received genetic confirmation of their diagnosis in case studies and cohort studies within this scoping review was relatively low. The majority of cohort studies (88/153; 58%) involved fewer than 50 participants. Based on the MMAT quality criteria for human studies, the majority of case studies (31/47; 66%) were of high quality, although most case studies did not meet criteria for inclusion of a representative sample. Cohort studies showed more variation in study quality (see Table 5 and further comments in the Discussion).

### Research question 5: which TAND levels have been investigated?

**Fig. 4:**
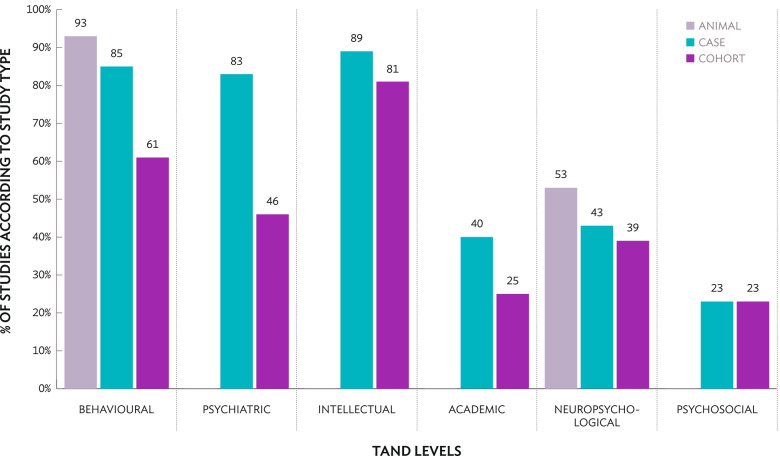
Research of different TAND levels based on study type (animal, case studies, cohort studies). The psychiatric, intellectual, academic and psychosocial levels were not applicable to animal studies and were therefore not shown

TAND level focus was analysed according to study type (see Fig. 4). The psychiatric, intellectual, academic and psychosocial levels of TAND were not relevant to animal studies. The majority of animal research focused on behavioural manifestations in mice and rats (28/30; 93%), such as social approach behaviours (e.g. [92]), anxiety (e.g. [93]), and social–communication behaviours associated with autism (e.g. [49]). Several animal studies focused on the neuropsychological level (16/30; 53%), as previously mentioned, particularly aspects relating to spatial processing, motor learning and memory (e.g. [94]). In human studies, both case and cohort studies predominantly reported on the behavioural (case studies: 40/47; 85%, cohort studies: 93/153; 61%) and intellectual levels (case studies: 42/47; 89%, cohort studies: 124/153; 81%).

Relatively few case or cohort studies reported academic (case studies: 19/47; 40%, cohort studies: 38/153; 25%), neuropsychological (case studies: 20/47; 43%, cohort studies: 60/153; 39%), or psychosocial information (case studies: 11/47; 23%, cohort studies: 35/153; 23%). It is important to note that although there were few psychosocial level studies overall, those that existed did specifically outline quality of life (e.g. [95]) or caregiver experiences (e.g. [96]) as a primary focus of the research, as evidenced by the study titles. By contrast, studies reporting academic information did not primarily focus on schooling or learning experiences of individuals with TSC. Academic information was briefly addressed as part of the larger participant or demographic information reporting, rather than as a primary research aim (e.g. [97]).

### Research question 6: which research methods and research measures have been used to investigate TAND?

A range of research methodologies have been used in TAND research to date (see Table 7). Animal studies exploring the behavioural and neuropsychological levels of TAND utilised a number of direct behavioural assessments (28/30; 93%) and neurobiological techniques in conjunction with behavioural assessments (23/30; 77%), such as immunohistochemistry and electroencephalography (EEG), to explore aspects relating to behaviour, learning and memory. Animal studies utilised standardised behavioural assessment protocols to explore aspects relating to behaviour and neuropsychological functioning. Such protocols included social versus inanimate object preference tests of social approach behaviours, marble burying to explore repetitive behaviour profiles, open-field tests of anxiety-related behaviours and ‘Morris water maze’ tasks to test spatial learning and memory processes.

**Table 7:** Research design and methodology of scoping review TAND studies

	Animal studies (***n*** = 30)	Case studies (***n*** = 47)	Cohort studies (***n*** = 153)
**Study design**^**a**^			
Quantitative	30 (100%)	12 (26%)	147 (96%)
Qualitative	N/A	45 (96%)	13 (9%)
*Descriptive*		42 (89%)	
*‘Typically qualitative’*		3 (6%)	
Control group (e.g. typically developing)	N/A	N/A	23 (15%)
Contrast group (e.g. genetic syndrome)	N/A	N/A	15 (10%)
Multiple control and contrast groups	N/A	N/A	5 (3%)
**Methodology**^**a**^			
Medical record review	N/A	41 (87%)	67 (44%)
Standardised questionnaires	N/A	3 (6%)	52 (34%)
Interviews (standardised/clinical/research)	N/A	0 (0%)	38 (25%)
Clinical report	N/A	1 (2%)	0 (0%)
Neuroimaging	23 (77%)	42 (89%)	74 (48%)
Direct neuropsychological assessment	N/A	12 (26%)	33 (22%)
IQ assessment	N/A	17 (36%)	80 (52%)
Direct behavioural assessment	28 (93%)	27 (57%)	9 (6%)
Physiological examination	14 (47%)	39 (83%)	35 (23%)
Diagnostic assessment	N/A	15 (32%)	51 (33%)
TAND Checklist	N/A	1 (2%)	6 (4%)
**Remote methodology**^**a**^			
Online survey	N/A	0 (0%)	9 (6%)
Telephone interview measures	N/A	0 (0%)	4 (3%)
Mobile application	N/A	0 (0%)	0 (0%)
**Intervention**^**a**^			
Non-pharmacological	15 (50%)	8 (17%)	0 (0%)
Pharmacological	24 (80%)	15 (32%)	8 (5%)
mTORi	14 (47%)	7 (15%)	5 (3%)
Other	4 (13%)	0 (0%)	4 (3%)

^a^Studies could span multiple data extraction points (e.g. a study that included an IQ assessment, direct behavioural assessment and neuroimaging); therefore, numbers and percentages reported within each category can exceed the maximum number of studies per study type. Percentages reported as percentage of total study type total (animal studies: *n* = 30, case studies: *n* = 47, cohort studies: *n* = 153). *N/A* information not applicable to study type

In human studies, case studies largely focused on medical record reviews of behaviour and psychiatric diagnoses (41/47; 87%), neuroimaging techniques such as EEG and MRI (42/47; 89%), and physiological examinations of tumour growth, seizures and physical health conditions in accordance with psychosocial manifestations (39/47; 83%). When exploring behavioural and intellectual aspects of TAND, case studies largely used retrospective or informant-report methodologies (e.g. case notes or caregiver report), as opposed to direct in-person methods of assessment (e.g. the Wechsler Adult Intelligence Scale, fourth edition [98]). It is important to note that few case studies provided specific details regarding the exact measures used, either from retrospective medical reviews, or when reporting on direct assessments (e.g. stating only ‘a comprehensive neuropsychology assessment’). Case studies also rarely utilised robust research tools, such as standardised questionnaires (3/47; 6%; [99–101]) or standardised interviews (0/47; 0%). The three case studies that utilised standardised questionnaires used measures such as the Aberrant Behaviour Checklist [102], the Social Responsiveness Scale, second edition [103] and the Behaviour Rating Inventory of Executive Function [104]. Only one case study included the TAND Checklist as an assessment measure [105].

Compared with case studies, cohort studies utilised more direct in-person methods of assessment, including direct IQ assessments (80/153; 52%), and diagnostic assessment tools (51/153; 33%). However, few cohort studies conducted any specific behavioural assessments (9/47; 6%). Of the nine studies identified [106–114], seven utilised screening measures for autism, such as the Autism Observation Scale for Infants (AOSI; [115]) and the Childhood Autism Rating Scale [116], two reported on specific behavioural items from the Autism Diagnostic Observation Schedule, second edition (ADOS-2; [117]), one involved a semantic decision task [96], and one explored musicality using a behavioural test battery [118]. Here, the ADOS-2 was considered a behavioural assessment as well as a diagnostic assessment when item-level analysis of the ADOS-2 was also conducted. The AOSI was considered a screening measure for ‘risk markers’ of autism in toddlers under the age of 18 months, before the age of typical autism diagnosis, and is therefore not considered a diagnostic instrument. Cohort studies largely utilised standardised questionnaires (52/153; 34%) and interviews (38/153; 25%), including standardised interviews such as the Vineland Adaptive Behaviour Scales, second edition [119], as well as clinical interviews and semi-structured research interviews (e.g. [120]). Cohort study diagnostic tools largely explored the profile of autism and psychiatric conditions. It is important to note that studies reporting on psychiatric comorbidities (e.g. [121, 122]) were largely based on psychiatric or clinical evaluation, and the specific details of diagnostic measures used were not provided. IQ measures included assessments such as the Bayley Scales of Infant Development, second edition [123] and the Wechsler Intelligence Scale for Children, third edition [124]. Since its publication in 2015, six cohort studies have utilised the TAND Checklist as an assessment measure [11, 19, 57, 61, 125, 126]. Three of these six studies reported data from the TOSCA registry [11, 19, 57].

### Research question 7: how much quantitative and qualitative TAND research has been conducted?

When referring to case studies, research was considered quantitative when within-group or follow-up descriptive statistics were reported. A distinction was made between case studies that were ‘typically qualitative’ (e.g. thematic analysis, focus groups) and case studies that were more ‘descriptive’ in nature (e.g. detailed family history, clinical opinion), as summarised in Table 7. The majority of case studies were ‘descriptive’ (42/47; 89%) as opposed to ‘typically qualitative’ (3/47; 6%). Of these, two included direct quotes from participants [127, 128], and one outlined qualitative themes based on a caregiver’s viewpoint [129]. Cohort studies were predominantly quantitative (147/153; 96%). Quantitative cohort studies were more likely to be descriptive single TSC cohort designs as opposed to experimental randomised control trials or between-group designs, as the majority of TAND cohort studies in this scoping review did not utilise contrast or control groups (110/153; 72%). Thirteen of the 153 cohort studies (9%) were qualitative [37, 54, 84, 120, 130–138]. Of these, only two included self-report interviews with individuals themselves with TSC, the others were qualitative perspectives of parents/caregivers [54, 134].

### Research question 8: how many intervention studies have been conducted?

A relatively high number of animal studies (26/30; 87%) and case studies (25/47; 53%) utilised interventions (see Table 7). Please note, data extraction only considered baseline reporting of TAND level information, not adverse effects or changes in TAND as a consequence of medication or treatment. Few cohort studies involved interventions (16/153; 11%). Of these 16 cohort intervention studies, five studies were everolimus clinical trials that explicitly considered TAND outcomes [50, 51, 82, 135, 139], five studies explored effects of antiepileptic medications [83, 140–143], two studies explored the effects of melatonin on sleep [144, 145], and four studies encompassed ‘other’ forms of intervention; the ketogenic diet [146], epilepsy surgery [147], ablation of tumours [148] and provision of information resources to aid parental understanding of TSC [138]. To date, no cohort studies have utilised non-pharmacological interventions in TSC.

### Research question 9: have remote technologies been utilised to study TAND?

Within human studies, the use of remote methodologies was low (see Table 7). No case studies utilised remote methods of data collection, compared with very few cohort studies (13/153; 8%). Of these 13 cohort studies, 11 studies specifically explored the psychosocial level of TAND in relation to caregiver experiences and quality of life variables, seven using online surveys [52, 90, 96, 131, 149–151] and four using telephone interviews [120, 134, 137, 147]. Two studies utilised online surveys to explore the behavioural and psychiatric levels of TAND, including sleep and behaviour in children [152] and mental health presentation and service provision in adults [153]. To date, no studies have utilised other technologies such as mobile applications, video conferencing, sensing technologies or robotics in TAND research.

### Research question 10: which TAND clusters have been studied?

Given the recent identification of natural TAND clusters, we were keen to determine the proportion of TAND research to date that has been performed in relation to these clusters. The TAND Checklist was only published in 2015 [13], and natural TAND clusters were only described in 2018 [61]. Therefore, it is important to note many publications in this scoping review may have used other terminologies that do not easily group into each of the seven natural TAND clusters. Evidently, there were relatively few publications that did not specifically fall into cluster groups across animal studies (0/30; 0%), case studies (2/47; 4%) or cohort studies (13/153; 8%). Across all study types, the majority of studies referenced multiple clusters per paper (animal studies = 20/30; 67%, case studies = 39/45; 87%, cohort studies = 95/140; 68%). The 45 cohort studies that explored only single clusters within each paper largely focused on the neuropsychological cluster (14/45; 31%) or the autism spectrum disorder–like cluster (19/45; 42%).

**Fig. 5:**
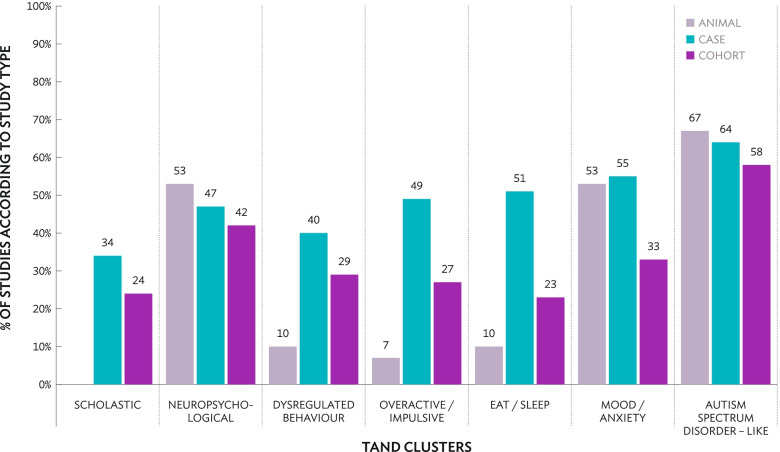
Research on different TAND clusters based on study type (animal, case studies, cohort studies). The scholastic cluster was not applicable to animal studies and was therefore not shown

As shown in Fig. 5, the majority of animal studies referenced the autism spectrum disorder–like cluster (20/30; 67%), the neuropsychological cluster (16/30; 53%) and the mood/anxiety cluster (16/30; 53%). Very few animal studies explored dysregulated behaviour (3/30; 10%; [48, 94, 154]), eat/sleep (3/30; 10%; [48, 154, 155]) or the overactive/impulsive clusters (2/30; 7%; [154, 156]). In human studies, both case studies (30/47; 64%) and cohort studies (88/153; 58%) largely focused on the autism spectrum disorder–like cluster, but infrequently referenced scholastic information (case studies: 16/47; 34%, cohort studies: 37/153; 24%). Across all TAND clusters, the eat/sleep cluster was the most under-researched in cohort studies (35/153; 23%).

## Discussion

The term ‘TAND’ was first coined in 2012 and published in 2013 [8] in an effort to reduce the clinical identification and treatment gap for the behavioural, psychiatric, intellectual, academic, neuropsychological and psychosocial manifestations associated with TSC, and to generate a ‘shared language’ that could aid global research efforts. This comprehensive scoping review was the first to synthesise the TAND research landscape using ten broad research questions. A summary of the findings and proposed directions for future research are outlined in Table 8. It was encouraging to see that there has been a clear increase in TAND research outputs in recent years, which may correspond with the adoption of ‘TAND’ terminology, and increased awareness of the TAND identification gap [8, 13, 14]. Despite the increase, a number of particular areas of TAND research warrant further investigation. In addition, the nature of a scoping review is to focus on ‘topline’ questions. To complement this review, in-depth content reviews within individual TAND clusters are warranted.

**Table 8:** Main findings and directions for future TAND research

Research question	Main findings	Directions for future research
1. TAND research across years	The number of TAND studies has increased over time, particularly since the term TAND was coined	Systematic reviews of each cluster should be conducted to capture TAND research that may have been missed
2. TAND research location	TAND research is predominantly conducted in HICs, the majority of HIC-led TAND research occurs in the USA and the UK	More research is needed particularly in South American, Middle Eastern, African, South-East Asia and Western Pacific countries
	TAND research is only evident in some LMICs due to representation from larger HIC-led multisite studies (e.g. Argentina, Mexico, Romania)	More TAND-focused research is needed that originates from LMICs reflecting specific cultural contexts, as opposed to research that only includes LMICs as part of larger multisite studies
3. Age distribution	The majority of human TAND research involves school-age and adolescent samples, with fewer studies including infant (0–3 years) or older adult samples (60 years and over)	Research that specifically focuses on the presentation of TAND across the lifespan and longitudinal research that investigates changes in TAND across developmental stages are needed
4. Study quality	Most case and cohort studies provide epilepsy and intellectual ability information but TSC genetic confirmation in studies is relatively low	TAND researchers should be encouraged to include data on seizures, intellectual ability and TSC genotype where possible
	The majority of TAND cohort studies involve fewer than 50 participants	Large-scale, coordinated cohort studies involving multisite international collaboration should be considered, except where specific research questions warrant small samples
	Animal studies and case studies are rated as high or relatively high quality; however, there is considerable variability in the quality of cohort studies, perhaps as a consequence of the quality appraisal tool used	Development of a scoping review quality appraisal tool will be beneficial to aid in the interpretation of study quality that allows for cross-comparison between different study designs
5. TAND levels	Animal studies largely report on the behavioural level of TAND	More contemporary animal research is needed that explores the neuropsychological level of TAND
	Case studies and cohort studies largely report on behavioural and intellectual TAND levels	In human studies, research is needed that focuses on the academic, neuropsychological and psychosocial levels of TAND
6. Research methods and measures	Animal studies utilise behavioural protocols and neuroimaging techniques	More TSC animal model research is needed that specifically utilises behavioural protocols that are relevant to TAND
	Case studies largely employ medical record reviews neuroimaging and physiological examinations, but rarely utilise standardised research assessments	TAND case studies would benefit from the inclusion of standardised assessments where possible and description of assessments completed as part of a clinical evaluation
	Cohort studies utilise IQ assessments and diagnostic research measures of autism	More direct behavioural assessments and behavioural observations in TAND research is needed, as well as a need to outline which specific diagnostic measures have been utilised when reporting TAND information based on psychological evaluation
	As a recently published measure, the TAND Checklist is rarely used as an assessment tool in human research	Human studies would benefit from the inclusion of TAND Checklist reporting where applicable
7. Quantitative and qualitative research	There are relatively few qualitative cohort studies	Qualitative research will be useful to refine the phenomenology of TAND behaviours that are not well-characterised in the existing literature
	The few qualitative studies that exist largely explore the psychosocial level	More qualititative research is needed that explores the under-researched areas of TAND
	The majority of quantitative cohort studies are descriptive as they do not utilise contrast or control groups	TAND cohort studies would benefit from the utilisation of appropriate control and contrast groups to determine TSC-specific manifestations of behaviours
8. Interventions	There are very few published intervention cohort studies, with a notable absence of any non-pharmacological intervention studies	Intervention studies are needed that take into account TAND outcomes, particularly exploring the effectiveness of non-pharamacological interventions in relation to TAND
9. Remote technologies	Only a small minority of cohort studies have employed remote methods of data collection, those that do largely explore the psychosocial level	Studies utilising remote technologies (e.g. mobile applications, video conferencing, online surveys) may help address TAND knowledge gaps and increase LMIC and population-based research
10. TAND clusters	A number of TAND clusters are under-researched in animal studies	More TSC animal models are needed that explore the dysregulated behaviour, overactive/impulsive and eat/sleep clusters
	Across human studies, the scholastic cluster is relatively under-researched	TAND clusters that are under-researched may be difficult to assess or quantify, and efforts should be made to evaluate existing assessments and tools to determine their utility in TSC cohorts
	The autism spectrum disorder–like cluster is the most widely researched across all three study types	Human studies would benefit from TAND-specific research that explores under-researched clusters, specifically the scholastic and eat/sleep clusters in TSC cohorts

Regarding the location of research, current TAND studies seems to over-represent TSC populations in HICs and may therefore not be generalisable or applicable to all global communities, such as in South America, the Middle East, Africa, and the Asia-Pacific region. In particular, very little research has been performed in LMICs. The socioeconomic, cultural and contextual factors that influence TAND identification and treatment in LMICs are therefore largely under-represented in the current TAND literature. Under-represented factors include but are not limited to: mental health service provision [157], access to autism diagnosis and screening assessments [158], disparities in the educational attainment of disability populations [159], and sociocultural differences in the epidemiology of eating disorders [160]. Where cohort studies included LMICs as part of international patient registries, it was unclear how much contribution LMICs had to study design or cultural translation, or the extent to which selection bias towards more severe/rare presentations of the disorder was evident [11]. Given that the majority of the world’s population live in LMICs, future research should place a particular emphasis on fostering LMIC-led TAND studies representative of the research needs and cultural context of individual countries.

In relation to the age distribution of TAND research participants, not many case studies described TAND level behaviours in infants, while cohort studies of infants mainly focused on autism presentation and related neurodevelopmental disability in relation to seizures. A broadening of infant research across TAND levels and clusters may therefore be a helpful next step. The number of case studies and cohort studies involving older adults was also low. From baseline data in the TOSCA registry, the oldest participant was 71 years old. However, it appears that most older adults living now with TSC may not have a confirmed genetic diagnosis [57] and therefore may not be represented within clinical population samples, or be identified within TSC clinics. The limited number of studies involving older adults may therefore reflect a broader issue with under-diagnosis of older individuals with rare genetic syndromes [161]. Given the longitudinal research indicating persistent TAND manifestations over time (e.g. [162, 163]), more research on TAND in older adults and across the developmental lifespan in general would be important. This includes examination of TAND associated with neurodegenerative processes, the relationship between changing seizure status over time and neurocognitive outcomes and whether an emergence or change in TSC physical characteristics (e.g. renal angiomyolipomas) is associated with TAND outcomes. This will be particularly important in relation to aspects of TAND that are under-researched, including the psychosocial level and eat/sleep cluster.

Study quality was difficult to establish, given the variability not only between study types (e.g. animal and human studies), but also between study designs (e.g. randomised controlled trials and quantitative descriptive cohort studies). Consequently, inter-rater agreement was low, particularly for cohort studies. It was also not possible to compare quality across cohort studies in a meaningful way, given that methodological criteria differed substantially according to study design (e.g. randomised controlled trials were rated according to five criteria that were non-comparable to the quantitative descriptive criteria). The absence of a ‘one-size-fits-all’ quality appraisal tool has long been recognised as a limitation in the scoping review field [64]. As such, very few scoping reviews endorse quality assessments [164], highlighting the need for a simplified quality appraisal tool that transcends multiple study designs and types. Small additions in TAND research protocols such as inclusion of seizure, IQ and genotype data, and generation of a core set of sociodemographic data could be helpful to increase study quality and determination thereof in future research. Development of large-scale international TAND consortia that uses high-quality, standardised measures have the potential to transform TAND research in the coming decades.

When examining research across TAND levels, it was clear that academic, neuropsychological and psychosocial aspects in individuals with TSC warrants further investigation. Given the very limited use of standardised assessment of specific learning skills (e.g. reading, writing, spelling, mathematics), an evaluation of suitable measures of scholastic ability (e.g. picture-based semantic association tests of vocabulary [165]) and reading and spelling (e.g. the Wechsler Objective Reading Dimensions test [166]) in diverse TSC populations, could be of significant value. Nevertheless, research on most TAND levels could benefit from improvements in rigour, for example: increased use of standardised behavioural ratings and observations, use of standardised psychiatric diagnostic schedules, IQ-based assessments in all individuals with TSC, standardisation of neuropsychological evaluations including ‘bedside’ neuropsychological assessments, and expansion of psychosocial research to differentiate between the impact of TSC and TAND on individuals and their caregivers.

Comparison of qualitative versus quantitative research in TAND exposed a disproportionately high representation of quantitative studies compared with qualitative studies across all TAND levels. Of the 13 qualitative studies included in this scoping review, eight considered the psychosocial level [54, 120, 131–134, 137, 138]. Of those, only one was derived from a LMIC (South Africa; [138]). Herein lies two directions for future research. First, more qualitative research overall is needed that explores quality of life variables, health-related burden and caregiver/sibling impact. Qualitative research that specifically aims to include the voices of individuals living themselves with TSC would be particularly valuable. Second, the psychosocial impact of a neurodevelopmental disorder is strongly influenced by contextual factors, which may include sociopolitical, cultural and economic contributors, amongst others. In order to ensure that we develop a global understanding of TAND, further qualitative research is required across HICs and LMICs [167].

Interventional research was very limited across case studies and cohort studies. Case studies mostly referenced the prescription of medications, most notably the mTORi everolimus. Examples of non-pharmacological interventions in case studies included cognitive behavioural therapy for obsessive compulsive disorder [127], ‘behavioural extinction’ for aggression [168] and ‘cognitive retraining’ techniques for mood, attention and behaviour [128]. In contrast, no cohort study in the review described non-pharmacological interventions. Following the completion of searches for this scoping review in March 2020, a protocol has since been published for a naturalistic developmental behavioural intervention (NDBI) for social communication in infants with TSC based on JASPER (Joint Attention, Symbolic Play, Engagement and Regulation [169, 170]), the results of which will significantly contribute to our understanding of intervention-based research for TAND. Future research should prioritise exploring the effectiveness of existing non-pharmacological interventions in TSC. This may be of particular value in the autism spectrum disorder–like cluster, where there is a strong evidence base for NDBIs, and within the dysregulated behaviour and mood/anxiety clusters, where existing cognitive/behavioural interventions have demonstrated marked success in individuals with autism [171, 172]. It should be noted that time and distance are significant barriers to enrolment in non-pharmacological intervention studies, as evident upon reflection of the JASPER TSC intervention [173].

In light of the COVID-19 pandemic, a move towards remote methods of assessment and intervention, as well as mobile health (mHealth) applications, demonstrates both practical and methodological sense. For this reason, we included a specific research question about the use of remote modalities. Our results showed a very limited number of studies that had used telephone interviews (e.g. [147]) and online surveys (e.g. [150]) for data collection. It was therefore very encouraging to see the innovative approach of the JASPER study outlined above using remote technologies for intervention delivery.

In an ongoing project (‘Empowering families through technology: a mobile-health project to reduce the TAND identification and treatment gap’) referred to as the TANDem project, the TAND consortium is aiming to address a number of TAND research gaps by using digital technologies. One of the key aims of the project is to develop a self-report quantified version of the TAND Checklist, the TAND-SQ, built into a ‘TAND toolkit’ mobile application (http://www.tandconsortium.org). We propose that significant resources should be allocated to the development of digital/mHealth applications that could facilitate TAND research (e.g. through remote data collection), research capacity-building (e.g. through building of a global network of TAND researchers), as well as leading to direct clinical impact through provision of training and fostering a shared language and understanding of TAND within clinician–patient relationships. Use of remote technologies could address many of the TAND research gaps identified in this scoping review, including low representation from LMICs, limited publication of qualitative TAND research and a need for non-pharmacological interventions involving TSC community populations.

The review gives weight to the complex and multifaceted nature of TSC, given that the majority of TAND studies were multi-cluster focused. Research therefore reflects the natural grouping of co-occurring TAND manifestations, and very few studies did not adhere to this seven-factor structure [63]. Those that did not correspond to an individual cluster (e.g. [174]) were studies that largely explored the intellectual level of TSC without reference to co-occurring behaviours or clinical conditions. Few studies focused on an individual cluster in specific detail, without reference to other clusters. As many TAND clusters are comparatively under-researched (e.g. scholastic, overactive/impulsive, eat/sleep), future TAND research may aim to specifically target these individual clusters for focused analysis. In animal research, investigation of dysregulated behaviours, eat/sleep, mood/anxiety and other neuropsychological skills (e.g. attention, dual tasking, planning) could provide valuable fundamental insights into the biological underpinnings and potential treatments within these TAND clusters. In human studies, highly under-explored clusters could employ qualitative designs as a first step towards refining phenomenology of behaviours that remain poorly understood in TSC, such as temper tantrums [175] or eating behaviours. Ensuring that the obvious research gaps in TAND clusters are filled with high-quality research in the coming decades will lead to significant improvements in clinical care for families and individuals who live with TSC and TAND.

### Limitations

We acknowledge a number of limitations in the current study. First, the inclusion or exclusion of studies at full-text level did not depend on any quality assessment. This step, which is in line with the scoping review methodology, might lead to a distorted view of the quality of evidence discussed here. Similarly, although we did not impose a language restriction on our searches, most of the resources found were written in English. These observations should primarily be understood as important findings of this scoping review and a key indication of the geographical distribution in relation to existing TAND research. We also did not determine whether the included manuscripts had a primary focus on TAND or whether TAND was a secondary or coincidental component of the work. Second, iterative search actions conducted after multiple consultations with other researchers or experts could have yielded other results than the consecutive search approach we utilised in the present study. Nevertheless, we are convinced that we have obtained the most comprehensive search results in the field to date to synthesise existing TAND research. Third, we are aware that our broad description and evaluation of the TAND research field lacks an in-depth discussion of the results. A more profound exploration of specific findings (e.g. an in-depth evaluation of animal study research in relation to TAND, a detailed analysis of standardised assessments employed by cohort studies, further consideration of specific TAND cluster items) may reveal other points for discussion or directions for future research. In this regard, this exploratory scoping review should be considered a roadmap for future TAND studies, including future reviews of the literature. Finally, as outlined in the Results section, there are several high profile or historical papers referenced in Table 1 that have not been captured by this scoping review. There are several reasons why their absence from the database searches may have occurred. Historical papers on online databases are primarily scanned hard copies from original journals, and therefore, electronic searches sometimes do not capture abstract content. Alternatively, it may be possible that search terms utilised here may not have reflected the historical language used by older studies (e.g. epiloia or Bourneville’s disease as synonyms for TSC), or that our current search terms were too stringent to capture more general descriptions of TSC neuropsychiatric conditions. We therefore recommend detailed cluster-based systematic reviews as a useful next step, with a particular emphasis and consideration on the inclusion of grey literature where appropriate.

## Conclusion

Although TAND research output has increased in recent years, significant gaps in knowledge remain. Overall, we observed an imbalance in TAND research across TAND levels and TAND clusters, with some levels (e.g. intellectual) and clusters (e.g. autism spectrum disorder–like cluster) much more widely researched than other aspects of TAND (e.g. scholastic skills and dysregulated behaviour). There is a clear need for future cohort studies that consider the presentation of TAND in older adult populations and qualitative methods to explore the phenomenology of behaviours that are poorly defined in TSC. Future research also needs to address the geographical disparities in TAND research that currently over-represents HIC involvement and under-represents LMICs. A move towards intervention is warranted, particularly non-pharmacological interventions that address TAND manifestations. The utilisation of specific remote methodologies, such as mobile applications and video conferencing technology will go some way to addressing several of the TAND research gaps identified here. International collaboration involving LMICs and utilisation of remote technologies are key aspects of the TANDem project, which aims to address the TAND identification and treatment gap in LMICs and alter the current TAND research landscape.

## Supplementary Information

**Additional file 1.** TSC and TAND level search terms.

**Additional file 2.** Two hundred thirty studies included in scoping review according to study type.

**Additional file 3.** Quality rating categorisation and grouping.

## Data Availability

All data generated and analysed for the purpose of this review are included in this publication and its supplementary information files.

## Abbreviations

ADHD: Attention deficit hyperactivity disorder
ADOS-2: Autism Diagnostic Observation Schedule, Second Edition
AOSI: Autism Observation Scale for Infants
ARRIVE: Animal Research Reporting of In Vivo Experiments
DSM-5: Diagnostic and Statistical Manual of Mental Disorders, Fifth Edition
EEG: Electroencephalography
EPISTOP: Long-term, Prospective Study Evaluating Clinical and Molecular Biomarkers of Epileptogenesis in a Genetic Model of Epilepsy—Tuberous Sclerosis Complex
GRIPP: Global regulator and integrator of a range of physiological processes
HIC: High-income country
ICD-11: International Classification of Diseases and Related Health Problems, Eleventh Edition
JASPER: Joint Attention Symbolic Play Engagement and Regulation
LMIC: Low–middle income country
mHealth: Mobile health
MMAT: Mixed Methods Appraisal Tool
MRI: Magnetic resonance imaging
mTOR: Mammalian/mechanistic target of rapamycin
mTORi: mTOR inhibitor
NDBI: Naturalistic developmental behavioural intervention
TAND: TSC-associated neuropsychiatric disorders
TANDem: Empowering families through technology: a mobile-health project to reduce the TAND identification and treatment gap
TOSCA: TuberOus SClerosis registry to increase disease Awareness
TSA: Tuberous Sclerosis Association
TSC: Tuberous sclerosis complex

## References

[1] Curatolo P, Moavero R, de Vries PJ (2015). Neurological and neuropsychiatric aspects of tuberous sclerosis complex. Lancet Neurol.

[2] Henske EP, Jóźwiak S, Kingswood JC, Sampson JR, Thiele EA (2016). Tuberous sclerosis complex. Nat Rev Dis Primers.

[3] Northrup H, Krueger DA, Roberds S, Smith K, Sampson J, Korf B (2013). Tuberous sclerosis complex diagnostic criteria update: recommendations of the 2012 International Tuberous Sclerosis Complex Consensus Conference. Pediatr Neurol.

[4] Rosset C, Netto CBO, Ashton-Prolla P (2017). TSC1 and TSC2 gene mutations and their implications for treatment in tuberous sclerosis complex: a review. Genet Mol Biol.

[5] Schwartz RA, Fernández G, Kotulska K, Jóźwiak S (2007). Tuberous sclerosis complex: advances in diagnosis, genetics, and management. J Am Acad Dermatol.

[6] Laplante M, Sabatini DM (2012). mTOR signaling in growth control and disease. Cell.

[7] Huber KM, Klann E, Costa-Mattioli M, Zukin RS (2015). Dysregulation of mammalian target of rapamycin signaling in mouse models of autism. J Neurosci.

[8] Krueger DA, Northrup H, Roberds S, Smith K, Sampson J, Korf B (2013). Tuberous sclerosis complex surveillance and management: recommendations of the 2012 International Tuberous Sclerosis Complex Consensus Conference. Pediatr Neurol.

[9] Mühlebner A, Bongaarts A, Sarnat H, Scholl T, Aronica E (2019). New insights into a spectrum of developmental malformations related to mTOR dysregulations: challenges and perspectives. J Anat.

[10] Crino PB, Nathanson KL, Henske EP (2006). The tuberous sclerosis complex. N Engl J Med.

[11] Marques R, Belousova E, Benedik MP, Carter T, Cottin V, Curatolo P (2019). Treatment patterns and use of resources in patients with tuberous sclerosis complex: insights from the TOSCA registry. Front Neurol.

[12] Northrup H, Aronow ME, Bebin EM, Bissler J, Darling TN, de Vries PJ (2021). Updated international tuberous sclerosis complex diagnostic criteria and surveillance and management recommendations. Pediatr Neurol.

[13] de Vries PJ, Whittemore VH, Leclezio L, Byars AW, Dunn D, Ess KC (2015). Tuberous sclerosis associated neuropsychiatric disorders (TAND) and the TAND Checklist. Pediatr Neurol.

[14] Leclezio L, de Vries P (2016). Towards an improved understanding of TSC-associated neuropsychiatric disorders (TAND). Adv Autism.

[15] Prather P, de Vries PJ (2004). Behavioral and cognitive aspects of tuberous sclerosis complex. J Child Neurol.

[16] de Vries PJ (2010). Targeted treatments for cognitive and neurodevelopmental disorders in tuberous sclerosis complex. Neurotherapeutics.

[17] Leclezio L, Jansen A, Whittemore VH, de Vries PJ (2015). Pilot validation of the tuberous sclerosis-associated neuropsychiatric disorders (TAND) checklist. Pediatr Neurol.

[18] Zöllner JP, Franz DN, Hertzberg C, Nabbout R, Rosenow F, Sauter M (2020). A systematic review on the burden of illness in individuals with tuberous sclerosis complex (TSC). Orphanet J Rare Dis.

[19] de Vries PJ, Belousova E, Benedik MP, Carter T, Cottin V, Curatolo P (2018). TSC-associated neuropsychiatric disorders (TAND): findings from the TOSCA natural history study. Orphanet J Rare Dis.

[20] Capal JK, Bernardino-Cuesta B, Horn PS, Murray D, Byars AW, Bing NM (2017). Influence of seizures on early development in tuberous sclerosis complex. Epilepsy Behav.

[21] Jansen AC, Vanclooster S, de Vries PJ, Fladrowski C, Beaure d'Augères G, Carter T (2020). Burden of illness and quality of life in tuberous sclerosis complex: findings from the TOSCA study. Front Neurol.

[22] Bourneville D (1880). Sclerose tubereuse der circonvolutions cerebrales: Idiotie et epilepsie hemiplegique. Arch Neurol.

[23] Vogt H (1908). Zur Pathologie und pathologischen Anatomie der verschiedenen Idiotieformen. Eur Neurol.

[24] Sherlock EB (1911). The feeble-minded: a guide to study and practice.

[25] Critchley M, Earl C (1932). Tuberose sclerosis and allied conditions. Brain.

[26] Lagos JC, Gomez MR (1967). Tuberous sclerosis: reappraisal of a clinical entity. Mayo Clin Proc.

[27] Hunt A (1983). Tuberous sclerosis: a survey of 97 cases. I: seizures, pertussis immunisation and handicap. Dev Med Child Neurol.

[28] Hunt A (1983). Tuberous sclerosis: a survey of 97 cases. II: physical findings. Dev Med Child Neurol.

[29] Hunt A (1983). Tuberous sclerosis: a survey of 97 cases. III: family aspects. Dev Med Child Neurol.

[30] Hunt A, Dennis J (1987). Psychiatric disorder among children with tuberous sclerosis. Dev Med Child Neurol.

[31] Hunt A, Shepherd C (1993). A prevalence study of autism in tuberous sclerosis. J Autism Dev Disord.

[32] Jambaqué I, Cusmai R, Curatolo P, Cortesi F, Perrot C, Dulac O (1991). Neuropsychological aspects of tuberous sclerosis in relation to epilepsy and MRI findings. Dev Med Child Neurol.

[33] Bolton PF, Griffiths PD (1997). Association of tuberous sclerosis of temporal lobes with autism and atypical autism. Lancet.

[34] Harrison JE, O'Callaghan FJ, Hancock E, Osborne JP, Bolton PF (1999). Cognitive deficits in normally intelligent patients with tuberous sclerosis. Am J Med Genet.

[35] Roach E, Gomez MR, Northrup H (1998). Tuberous sclerosis complex consensus conference: revised clinical diagnostic criteria. J Child Neurol.

[36] Roach E, DiMario FJ, Kandt RS, Northrup H (1999). Tuberous sclerosis consensus conference: recommendations for diagnostic evaluation. J Child Neurol.

[37] O’Callaghan F, Harris T, Joinson C, Bolton P, Noakes M, Presdee D (2004). The relation of infantile spasms, tubers, and intelligence in tuberous sclerosis complex. Arch Dis Child.

[38] Zaroff CM, Barr WB, Carlson C, LaJoie J, Madhavan D, Miles DK (2006). Mental retardation and relation to seizure and tuber burden in tuberous sclerosis complex. Seizure.

[39] de Vries P, Humphrey A, McCartney D, Prather P, Bolton P, Hunt A (2005). Consensus clinical guidelines for the assessment of cognitive and behavioural problems in tuberous sclerosis. Eur Child Adolesc Psychiatry.

[40] de Vries PJ, Hunt A, Bolton PF (2007). The psychopathologies of children and adolescents with tuberous sclerosis complex (TSC). Eur Child Adolesc Psychiatry.

[41] Kopp CM, Muzykewicz DA, Staley BA, Thiele EA, Pulsifer MB (2008). Behavior problems in children with tuberous sclerosis complex and parental stress. Epilepsy Behav.

[42] Staley BA, Montenegro MA, Major P, Muzykewicz DA, Halpern EF, Kopp CM (2008). Self-injurious behavior and tuberous sclerosis complex: frequency and possible associations in a population of 257 patients. Epilepsy Behav.

[43] Tee AR, Manning BD, Roux PP, Cantley LC, Blenis J (2003). Tuberous sclerosis complex gene products, Tuberin and Hamartin, control mTOR signaling by acting as a GTPase-activating protein complex toward Rheb. Curr Biol.

[44] Zhang H, Cicchetti G, Onda H, Koon HB, Asrican K, Bajraszewski N (2003). Loss of Tsc1/Tsc2 activates mTOR and disrupts PI3K-Akt signaling through downregulation of PDGFR. J Clin Investig.

[45] de Vries PJ, Howe CJ (2007). The tuberous sclerosis complex proteins–a GRIPP on cognition and neurodevelopment. Trends Mol Med.

[46] Curatolo P, Moavero R (2012). mTOR inhibitors in tuberous sclerosis complex. Curr Neuropharmacol.

[47] Davies DM, de Vries PJ, Johnson SR, McCartney DL, Cox JA, Serra AL (2011). Sirolimus therapy for angiomyolipoma in tuberous sclerosis and sporadic lymphangioleiomyomatosis: a phase 2 trial. Clin Cancer Res.

[48] Ehninger D, Han S, Shilyansky C, Zhou Y, Li W, Kwiatkowski DJ (2008). Reversal of learning deficits in a Tsc2+/− mouse model of tuberous sclerosis. Nat Med.

[49] Tsai PT, Greene-Colozzi E, Goto J, Anderl S, Kwiatkowski DJ, Sahin M (2013). Prenatal rapamycin results in early and late behavioral abnormalities in wildtype C57BL/6 mice. Behav Genet.

[50] Krueger DA, Sadhwani A, Byars AW, de Vries PJ, Franz DN, Whittemore VH (2017). Everolimus for treatment of tuberous sclerosis complex-associated neuropsychiatric disorders. Ann Clin Transl Neurol.

[51] Overwater IE, Rietman AB, Mous SE, Bindels-de Heus K, Rizopoulos D, Leontine W (2019). A randomized controlled trial with everolimus for IQ and autism in tuberous sclerosis complex. Neurology.

[52] Skalicky AM, Rentz AM, Liu Z, Said Q, Nakagawa JA, Frost MD (2018). Economic burden, work, and school productivity in individuals with tuberous sclerosis and their families. J Med Econ.

[53] Vekeman F, Magestro M, Karner P, Duh MS, Nichols T, van Waalwijk van Doorn-Khosrovani SB (2015). Kidney involvement in tuberous sclerosis complex: the impact on healthcare resource use and costs. J Med Econ..

[54] Both P, Ten Holt L, Mous S, Patist J, Rietman A, Dieleman G (2018). Tuberous sclerosis complex: concerns and needs of patients and parents from the transitional period to adulthood. Epilepsy Behav.

[55] Zöllner JP, Conradi N, Sauter M, Knuf M, Knake S, Kurlemann G (2021). Quality of life and its predictors in adults with tuberous sclerosis complex (TSC): a multicentre cohort study from Germany. Neurol Res Pract.

[56] Kingswood JC, Bruzzi P, Curatolo P, de Vries PJ, Fladrowski C, Hertzberg C (2014). TOSCA–first international registry to address knowledge gaps in the natural history and management of tuberous sclerosis complex. Orphanet J Rare Dis.

[57] Kingswood JC, d’Augères GB, Belousova E, Ferreira JC, Carter T, Castellana R (2017). TuberOus SClerosis registry to increase disease Awareness (TOSCA)–baseline data on 2093 patients. Orphanet J Rare Dis.

[58] Sahin M, Henske EP, Manning BD, Ess KC, Bissler JJ, Klann E (2016). Advances and future directions for tuberous sclerosis complex research: recommendations from the 2015 strategic planning conference. Pediatr Neurol.

[59] American Psychological Association (2013). Diagnostic and Statistical Manual of Mental Disorders.

[60] World Health Organisation (2018). The ICD-11 Classification of Mental and Behavioral Disorders: diagnostic criteria for research.

[61] Leclezio L, Gardner-Lubbe S, de Vries PJ (2018). Is it feasible to identify natural clusters of TSC-associated neuropsychiatric disorders (TAND)?. Pediatr Neurol.

[62] de Vries PJ, Belousova E, Benedik MP, Carter T, Cottin V, Curatolo P (2020). Natural clusters of tuberous sclerosis complex (TSC)-associated neuropsychiatric disorders (TAND): new findings from the TOSCA TAND research project. J Neurodev Disord.

[63] de Vries PJ, Leclezio L, Gardner-Lubbe S, Krueger D, Sahin M, Sparagana S (2021). Multivariate data analysis identifies natural clusters of tuberous sclerosis complex associated neuropsychiatric disorders (TAND). Orphanet J Rare Dis.

[64] Levac D, Colquhoun H, O’Brien KK (2010). Scoping studies: advancing the methodology. Implement Sci.

[65] Anderson S, Allen P, Peckham S, Goodwin N (2008). Asking the right questions: scoping studies in the commissioning of research on the organisation and delivery of health services. Health Res Policy Syst.

[66] Davis K, Drey N, Gould D (2009). What are scoping studies? A review of the nursing literature. Int J Nurs Stud.

[67] Arksey H, O'Malley L (2005). Scoping studies: towards a methodological framework. Int J Soc Res Methodol.

[68] Hopewell S, McDonald S, Clarke M, Egger M (2007). Grey literature in meta-analyses of randomized trials of health care interventions. Cochrane Database Syst Rev.

[69] Paez A (2017). Gray literature: an important resource in systematic reviews. J Evid Based Med.

[70] Tricco AC, Lillie E, Zarin W, O’Brien K, Colquhoun H, Kastner M (2016). A scoping review on the conduct and reporting of scoping reviews. BMC Med Res Methodol.

[71] Adams J, Hillier-Brown FC, Moore HJ, Lake AA, Araujo-Soares V, White M (2016). Searching and synthesising ‘grey literature’ and ‘grey information’ in public health: critical reflections on three case studies. Syst Rev.

[72] du Sert NP, Hurst V, Ahluwalia A, Alam S, Avey MT, Baker M (2020). The ARRIVE guidelines 2.0: updated guidelines for reporting animal research. J Cereb Blood Flow Metab.

[73] Hong QN, Fàbregues S, Bartlett G, Boardman F, Cargo M, Dagenais P (2018). The Mixed Methods Appraisal Tool (MMAT) version 2018 for information professionals and researchers. Educ Inf.

[74] Fryer AE, Osborne JP, Tan R, Siggers DC (1987). Tuberous sclerosis: a large family with no history of seizures or mental retardation. J Med Genet.

[75] Lawlor BA, Maurer RG (1987). Tuberous sclerosis and the autistic syndrome. Br J Psychiatry.

[76] Oliver BE (1987). Tuberous sclerosis and autistic syndrome. Br J Psychiatry.

[77] Webb DW, Fryer AE, Osborne JP (1991). On the incidence of fits and mental retardation in tuberous sclerosis. J Med Genet.

[78] Webb DW, Thomson J, Osborne J (1991). Cranial magnetic resonance imaging in patients with tuberous sclerosis and normal intellect. Arch Dis Child.

[79] Von Der Brelie C, Waltereit R, Zhang L, Beck H, Kirschstein T (2006). Impaired synaptic plasticity in a rat model of tuberous sclerosis. Eur J Neurosci.

[80] Waltereit R, Welzl H, Dichgans J, Lipp HP, Schmidt WJ, Weller M (2006). Enhanced episodic-like memory and kindling epilepsy in a rat model of tuberous sclerosis. J Neurochem.

[81] World Bank Group. World Bank list of economies. Washington, DC; 2020. Retrieved from: http://databank.worldbank.org/data/download/site-content/CLASS.xlsx

[82] de Vries PJ, Franz DN, Curatolo P, Nabbout R, Neary M, Herbst F (2018). Measuring health-related quality of life in tuberous sclerosis complex - Psychometric evaluation of three instruments in individuals with refractory epilepsy. Front Pharmacol.

[83] Cusmai R, Moavero R, Bombardieri R, Vigevano F, Curatolo P (2011). Long-term neurological outcome in children with early-onset epilepsy associated with tuberous sclerosis. Epilepsy Behav.

[84] Gillberg JC, Gillberg C, Ahlsén G (1994). Autistic behaviour and attention deficits in tuberous sclerosis: a population-based study. Dev Med Child Neurol.

[85] Joinson C, O'Callaghan F, Osborne J, Martyn C, Harris T, Bolton P (2003). Learning disability and epilepsy in an epidemiological sample of individuals with tuberous sclerosis complex. Psychol Med.

[86] Kingswood C, Bolton P, Crawford P, Harland C, Johnson SR, Sampson JR (2016). The clinical profile of tuberous sclerosis complex (TSC) in the United Kingdom: a retrospective cohort study in the Clinical Practice Research Datalink (CPRD). Eur J Pediatr Neurol.

[87] Lennert B, Farrelly E, Sacco P, Pira G, Frost M (2013). Resource utilization in children with tuberous sclerosis complex and associated seizures: a retrospective chart review study. J Child Neurol.

[88] Shepherd C, Koepp M, Myland M, Patel K, Miglio C, Siva V (2017). Understanding the health economic burden of patients with tuberous sclerosis complex (TSC) with epilepsy: a retrospective cohort study in the UK Clinical Practice Research Datalink (CPRD). BMJ Open.

[89] Tye C, Thomas LE, Sampson JR, Lewis J, O'Callaghan F, Yates JR (2018). Secular changes in severity of intellectual disability in tuberous sclerosis complex: a reflection of improved identification and treatment of epileptic spasms?. Epilepsia Open.

[90] Tritton T, Bennett B, Brohan E, Grant L, Cooper A, Fladrowski C (2019). Health utilities and quality of life in individuals with tuberous sclerosis complex (TSC) who experience epileptic seizures: a web-based survey. Epilepsy Behav.

[91] Bhattacharya A, Das S, Nath K, Dutta D, Saddichha S (2012). Atypical presentation of tuberous sclerosis and obsessive compulsive disorder in an adult male. Ann Indian Acad Neurol.

[92] Ehninger D, Sano Y, de Vries PJ, Dies K, Franz D, Geschwind DH (2012). Gestational immune activation and Tsc2 haploinsufficiency cooperate to disrupt fetal survival and may perturb social behavior in adult mice. Mol Psychiatry.

[93] Zeng L-H, Ouyang Y, Gazit V, Cirrito JR, Jansen LA, Ess KC (2007). Abnormal glutamate homeostasis and impaired synaptic plasticity and learning in a mouse model of tuberous sclerosis complex. Neurobiol Dis.

[94] Kosillo P, Doig NM, Ahmed KM, Agopyan-Miu AH, Wong CD, Conyers L (2019). Tsc1-mTORC1 signaling controls striatal dopamine release and cognitive flexibility. Nat Commun.

[95] Bar C, Ghobeira R, Azzi R, Ville D, Riquet A, Touraine R (2019). Experience of follow-up, quality of life, and transition from pediatric to adult healthcare of patients with tuberous sclerosis complex. Epilepsy Behav.

[96] Rentz AM, Skalicky AM, Pashos CL, Liu Z, Magestro M, Pelletier CL (2015). Caring for children with tuberous sclerosis complex: what is the physical and mental health impact on caregivers?. J Child Neurol.

[97] Vignoli A, Briola FL, Turner K, Scornavacca G, Chiesa V, Zambrelli E (2013). Epilepsy in TSC: certain etiology does not mean certain prognosis. Epilepsia.

[98] Wechsler D (2008). Wechsler adult intelligence scale.

[99] Ishii R, Wataya-Kaneda M, Canuet L, Nonomura N, Nakai Y, Takeda M (2015). Everolimus improves behavioral deficits in a patient with autism associated with tuberous sclerosis: a case report. Neuropsychiatr Electrophysiol.

[100] Vlaskamp C, Poil S-S, Jansen F, Linkenkaer-Hansen K, Durston S, Oranje B (2017). Bumetanide as a candidate treatment for behavioral problems in tuberous sclerosis complex. Front Neurol.

[101] Yui K, Imataka G, Sasaki H, Kawasaki Y, Okanshi T, Shiroki R (2019). Improvement in impaired social cognition but not seizures by everolimus in a child with tuberous sclerosis-associated autism through increased serum antioxidant proteins and oxidant/antioxidant status. Case Rep Pediatr.

[102] Aman MG, Singh NN, Stewart AW, Field CJ (1985). The Aberrant Behavior Checklist: a behavior rating scale for the assessment of treatment effects. Am J Ment Defic.

[103] Constantino JN, Gruber CP (2012). Social responsiveness scale.

[104] Gioia GA, Isquith PK, Guy SC, Kenworthy L (2000). Behavior rating inventory of executive function.

[105] Waszak PM, Lewandowska K, Kasprzycka-Waszak W, Gordon W, Zagożdżon P (2017). Tuberous sclerosis-associated neuropsychiatric disorders-case report. Neuropsychiatr Neuropsychol.

[106] Capal JK, Horn PS, Murray DS, Byars AW, Bing NM, Kent B (2017). Utility of the Autism Observation Scale for Infants in early identification of autism in tuberous sclerosis complex. Pediatr Neurol.

[107] Gallagher A, Tanaka N, Suzuki N, Liu H, Thiele EA, Stufflebeam SM (2013). Diffuse cerebral language representation in tuberous sclerosis complex. Epilepsy Res.

[108] Jeste SS, Varcin KJ, Hellemann GS, Gulsrud AC, Bhatt R, Kasari C (2016). Symptom profiles of autism spectrum disorder in tuberous sclerosis complex. Neurology.

[109] Jeste SS, Wu JY, Senturk D, Varcin K, Ko J, McCarthy B (2014). Early developmental trajectories associated with ASD in infants with tuberous sclerosis complex. Neurology.

[110] Lewis J, Thomas H, Murphy K, Sampson J (2004). Genotype and psychological phenotype in tuberous sclerosis. J Med Genet.

[111] Matsuyama K, Ohsawa I, Ogawa T (2007). Do children with tuberous sclerosis complex have superior musical skill? A unique tendency of musical responsiveness in children with TSC. Med Sci Monit.

[112] McDonald NM, Varcin KJ, Bhatt R, Wu JY, Sahin M, Nelson CA (2017). Early autism symptoms in infants with tuberous sclerosis complex. Autism Res.

[113] Scherrer B, Prohl AK, Taquet M, Kapur K, Peters JM, Tomas-Fernandez X (2020). The connectivity fingerprint of the fusiform gyrus captures the risk of developing autism in infants with tuberous sclerosis complex. Cereb Cortex.

[114] Schoenberger A, Capal JK, Ondracek A, Horn PS, Murray D, Byars AW (2020). Language predictors of autism spectrum disorder in young children with tuberous sclerosis complex. Epilepsy Behav.

[115] Bryson SE, Zwaigenbaum L (2014). Autism Observation Scale for Infants. Comprehensive guide to autism.

[116] Schopler E, Reichler RJ, DeVellis RF, Daly K (1980). Toward objective classification of childhood autism: Childhood Autism Rating Scale (CARS). J Autism Dev Disord.

[117] Lord C, Rutter M, DiLavore PC, Risi S, Gotham K, Bishop S (2012). Autism diagnostic observation schedule manual.

[118] Matsuyama K (2005). Correlation between musical responsiveness and developmental age among early age children as assessed by the Non-Verbal Measurement of the Musical Responsiveness of Children. Med Sci Monit.

[119] Sparrow SS, Cicchetti DV, Balla DA (2005). Vineland adaptive behavior scales.

[120] Whitehead LC, Gosling V (2003). Parent’s perceptions of interactions with health professionals in the pathway to gaining a diagnosis of tuberous sclerosis (TS) and beyond. Res Dev Disabil.

[121] Chung TK, Lynch ER, Fiser CJ, Nelson DA, Tudor C, Franz DN (2011). Psychiatric comorbidity and treatment response in patients with tuberous sclerosis complex. Ann Clin Psychiatry.

[122] Muzykewicz DA, Newberry P, Danforth N, Halpern EF, Thiele EA (2007). Psychiatric comorbid conditions in a clinic population of 241 patients with tuberous sclerosis complex. Epilepsy Behav.

[123] Bayley N, Reuner G (1969). Bayley scales of infant development.

[124] Wechsler D (1991). Wechsler intelligence scale for children.

[125] Ebrahimi-Fakhari D, Hussong J, Flotats-Bastardas M, Ebrahimi-Fakhari D, Zemlin M, von Gontard A (2019). Tuberous sclerosis complex associated neuropsychiatric disorders and parental stress: findings from a national, prospective TSC surveillance study. Neuropediatrics.

[126] Toldo I, Brasson V, Miscioscia M, Pelizza MF, Manara R, Sartori S (2019). Tuberous sclerosis-associated neuropsychiatric disorders: a paediatric cohort study. Dev Med Child Neurol.

[127] Ozgur BG, Aksu H, Tosun AF (2018). Comorbid obsessive compulsive disorder in a child with tuberous sclerosis complex. Psychiatry Behav Sci.

[128] Sharma P, Rao K (2002). Psychological intervention in tuberous sclerosis: a case report. Indian J Psychiatry.

[129] Willacy H (2016). The impact of tuberous sclerosis complex–a parent’s perspective. Adv Autism.

[130] Goh S, Kwiatkowski DJ, Dorer DJ, Thiele EA (2005). Infantile spasms and intellectual outcomes in children with tuberous sclerosis complex. Neurology.

[131] Graffigna G, Bosio C, Cecchini I (2013). Assisting a child with tuberous sclerosis complex (TSC): a qualitative deep analysis of parents’ experience and caring needs. BMJ Open.

[132] Hunt A (1998). A comparison of the abilities, health and behaviour of 23 people with tuberous sclerosis at age 5 and as adults. J Appl Res Intellect Disabil.

[133] Hunt A, Stores G (1994). Sleep disorder and epilepsy in children with tuberous sclerosis: a questionnaire-based study. Dev Med Child Neurol.

[134] McDonald A, Goodwin J, Roberts S, Fish L, Vaughan B, Cooper A (2019). ‘We’ve made the best of it. But we do not have a normal life’: families’ experiences of tuberous sclerosis complex and seizure management. J Intellect Disabil Res.

[135] Mizuguchi M, Ikeda H, Kagitani-Shimono K, Yoshinaga H, Suzuki Y, Aoki M (2019). Everolimus for epilepsy and autism spectrum disorder in tuberous sclerosis complex: EXIST-3 substudy in Japan. Brain Dev.

[136] Morrison PJ, O’Neill T, Hardy R, Shepherd CW, Donnelly DE (2015). The prevalence of pica in tuberous sclerosis complex. SpringerPlus.

[137] Parker M (1996). Families caring for chronically ill children with tuberous sclerosis complex. Fam Community Health.

[138] Samia P, Donald KA, Schlegel B, Wilmshurst JM (2015). Parental understanding of tuberous sclerosis complex. J Child Neurol.

[139] Krueger DA, Wilfong AA, Holland-Bouley K, Anderson AE, Agricola K, Tudor C (2013). Everolimus treatment of refractory epilepsy in tuberous sclerosis complex. Ann Neurol.

[140] Bombardieri R, Pinci M, Moavero R, Cerminara C, Curatolo P (2010). Early control of seizures improves long-term outcome in children with tuberous sclerosis complex. Eur J Pediatr Neurol.

[141] Jambaqué I, Chiron C, Dumas C, Mumford J, Dulac O (2000). Mental and behavioural outcome of infantile epilepsy treated by vigabatrin in tuberous sclerosis patients. Epilepsy Res.

[142] Jóźwiak S, Kotulska K, Domańska-Pakieła D, Łojszczyk B, Syczewska M, Chmielewski D (2011). Antiepileptic treatment before the onset of seizures reduces epilepsy severity and risk of mental retardation in infants with tuberous sclerosis complex. Eur J Pediatr Neurol.

[143] Yum M-S, Lee EH, Ko T-S (2013). Vigabatrin and mental retardation in tuberous sclerosis: infantile spasms versus focal seizures. J Child Neurol.

[144] Hancock E, O'Callaghan F, Osborne JP (2005). Effect of melatonin dosage on sleep disorder in tuberous sclerosis complex. J Child Neurol.

[145] O'Callaghan F, Clarke A, Hancock E, Hunt A, Osborne J (1999). Use of melatonin to treat sleep disorders in tuberous sclerosis. Dev Med Child Neurol.

[146] Kossoff EH, Thiele EA, Pfeifer HH, McGrogan JR, Freeman JM (2005). Tuberous sclerosis complex and the ketogenic diet. Epilepsia.

[147] Roth J, Olasunkanmi A, MacAllister WS, Weil E, Uy CC, Devinsky O (2011). Quality of life following epilepsy surgery for children with tuberous sclerosis complex. Epilepsy Behav.

[148] Ierardi AM, Petrillo M, Coppola A, Angileri SA, Galassi A, Padovano B (2019). Percutaneous microwave ablation of renal angiomyolipomas in tuberous sclerosis complex to improve the quality of life: preliminary experience in an Italian center. Radiol Med.

[149] Rentz AM, Skalicky AM, Liu Z, Wheless JW, Dunn DW, Frost MD (2015). Tuberous sclerosis complex: a survey of health care resource use and health burden. Pediatr Neurol.

[150] Rentz AM, Skalicky AM, Liu Z, Dunn DW, Frost MD, Nakagawa JA (2018). Burden of renal angiomyolipomas associated with tuberous sclerosis complex: results of a patient and caregiver survey. J Paitent Rep Outcomes.

[151] Skalicky AM, Rentz AM, Liu Z, Wheless JW, Pelletier CL, Dunn DW (2015). The burden of subependymal giant cell astrocytomas associated with tuberous sclerosis complex: results of a patient and caregiver survey. J Child Neurol.

[152] Trickett J, Heald M, Oliver C, Richards C (2018). A cross-syndrome cohort comparison of sleep disturbance in children with Smith-Magenis syndrome, Angelman syndrome, autism spectrum disorder and tuberous sclerosis complex. J Neurodev Disord.

[153] Mowrey KE, Ashfaq M, Pearson DA, Hashmi SS, Roberds SL, Farach LS (2019). The impact of psychiatric symptoms on tuberous sclerosis complex and utilization of mental health treatment. Pediatr Neurol.

[154] Carson RP, Fu C, Winzenburger P, Ess KC (2013). Deletion of Rictor in neural progenitor cells reveals contributions of mTORC2 signaling to tuberous sclerosis complex. Hum Mol Genet.

[155] Zhang B, Guo D, Han L, Rensing N, Satoh A, Wong M (2020). Hypothalamic orexin and mechanistic target of rapamycin activation mediate sleep dysfunction in a mouse model of tuberous sclerosis complex. Neurobiol Dis.

[156] Kelly E, Schaeffer SM, Dhamne SC, Lipton JO, Lindemann L, Honer M (2018). mGluR5 modulation of behavioral and epileptic phenotypes in a mouse model of tuberous sclerosis complex. Neuropsychopharmacology.

[157] Rathod S, Pinninti N, Irfan M, Gorczynski P, Rathod P, Gega L (2017). Mental health service provision in low-and middle-income countries. Health Serv Insights.

[158] Daley TC, Singhal N, Krishnamurthy V (2013). Ethical considerations in conducting research on autism spectrum disorders in low and middle income countries. J Autism Dev Disord.

[159] Mizunoya S, Mitra S, Yamasaki I (2018). Disability and school attendance in 15 low-and middle-income countries. World Dev.

[160] Hoek HW (2016). Review of the worldwide epidemiology of eating disorders. Curr Opin Psychiatry.

[161] Claussnitzer M, Cho JH, Collins R, Cox NJ, Dermitzakis ET, Hurles ME (2020). A brief history of human disease genetics. Nature.

[162] van Eeghen AM, Chu-Shore CJ, Pulsifer MB, Camposano SE, Thiele EA (2012). Cognitive and adaptive development of patients with tuberous sclerosis complex: a retrospective, longitudinal investigation. Epilepsy Behav.

[163] Wilde L, Wade K, Eden K, Moss J, de Vries P, Oliver C (2018). Persistence of self-injury, aggression and property destruction in children and adults with tuberous sclerosis complex. J Intellect Dis Res.

[164] Pham MT, Rajić A, Greig JD, Sargeant JM, Papadopoulos A, McEwen SA (2014). A scoping review of scoping reviews: advancing the approach and enhancing the consistency. Res Synth Methods.

[165] Laws G, Briscoe J, Ang S-Y, Brown H, Hermena E, Kapikian A (2015). Receptive vocabulary and semantic knowledge in children with SLI and children with Down syndrome. Child Neuropsychol.

[166] Rust J, Golombok S, Trickey G (1993). WORD, Wechsler Objective Reading Dimensions manual.

[167] McKenzie J, McConkey R (2016). Caring for adults with intellectual disability: the perspectives of family carers in South Africa. J Appl Res Intellect Disabil.

[168] Gipson TT, Jennett H, Wachtel L, Gregory M, Poretti A, Johnston MV (2013). Everolimus and intensive behavioral therapy in an adolescent with tuberous sclerosis complex and severe behavior. Epilepsy Behav Case Rep.

[169] Kasari C, Gulsrud A, Paparella T, Hellemann G, Berry K (2015). Randomized comparative efficacy study of parent-mediated interventions for toddlers with autism. J Consult Clin Psychol.

[170] McDonald NM, Hyde C, Choi AB, Gulsrud AC, Kasari C, Nelson CA (2020). Improving developmental abilities in infants with tuberous sclerosis complex: a pilot behavioral intervention study. Infants Young Child.

[171] Rodgers J, Goodwin J, Parr JR, Grahame V, Wright C, Padget J (2019). Coping with Uncertainty in Everyday Situations (CUES©) to address intolerance of uncertainty in autistic children: study protocol for an intervention feasibility trial. Trials.

[172] Singh NN, Lancioni GE, Manikam R, Winton AS, Singh AN, Singh J (2011). A mindfulness-based strategy for self-management of aggressive behavior in adolescents with autism. Res Autism Spectr Disord.

[173] Hyde C, Pizzano M, McDonald NM, Nelson CA, Kasari C, Thiele EA (2020). A telehealth approach to improving clinical trial access for infants with tuberous sclerosis complex. J Neurodev Disord.

[174] Overwater IE, Verhaar BJ, Lingsma HF, Bindels-de Heus GC, van den Ouweland AM, Nellist M (2017). Interdependence of clinical factors predicting cognition in children with tuberous sclerosis complex. J Neurol.

[175] Al-Busaidi ZQ (2008). Qualitative research and its uses in health care. Sultan Qaboos Univ Med J.

