# Supplementary material for: The research landscape of tuberous sclerosis complex–associated neuropsychiatric disorders (TAND)—a comprehensive scoping review
Source: J Neurodev Disord. 2022 Feb 13;14:13. doi: 10.1186/s11689-022-09423-3 (PMC8853020; doi:10.1186/s11689-022-09423-3)
Supplement: Supplementary file 1 — Additional file 1. TSC and TAND level search terms. [file 11689_2022_9423_MOESM1_ESM.pdf]

Additional File 1. TSC and TAND level search terms

TSC Search Terms	TAND Level Search Terms[1]
Tuberous sclerosis<br>Tuberous sclerosis complex<br>TS<br>TSC<br>TSC-Associated Neuropsychiatric Disorders<br>TAND	*Behavioural level*<br>"anxiety" OR "depression" OR "mood difficulties" OR "aggression" OR "temper tantrums" OR "self-injury" OR "social difficulties" OR "social deficits" OR "communication difficulties" OR "peer* difficulties" OR "relationship difficulties" OR "speech difficulties" OR "dysregulated behavio*" OR "behavio* difficulties" OR "hyperactivity" OR "overactivity" OR "impulsivity" OR "restless*" OR "sleep* difficulties" OR "eat* difficulties"<br>*Psychiatric level*<br>"neuro*" OR "psychiatric disorder" OR "autism spectrum disorder" OR "ASD" OR "autism" OR "Asperger syndrome" OR "pervasive developmental disorder" OR "PDD" OR "development* difficulties" OR "attention" OR "attention deficit disorder" OR "ADD" OR "attention deficit hyperactivity disorder" OR "ADHD" OR "anxiety disorder" OR "depressive disorder" OR "mood disorder" OR "obsessive compulsive disorder" OR "OCD" OR "psychosis" OR "psychotic disorder"<br>*Intellectual level*<br>"intellectual dis*" OR "intellectual ability" OR "cognitive dys*" OR "cognitive function*" OR "mental retardation"<br>*Academic level*<br>"school difficulties," OR "scholastic difficulties" OR "education* difficulties" OR "academic difficulties" OR "learning difficulties" OR "reading difficulties" OR "writing difficulties" OR "spelling difficulties" OR "mathematics difficulties"<br>*Neuropsychological level*<br>"neuropsychological function*" OR "neuropsychological difficulties" OR "attention* difficulties" OR "concentration difficulties" OR "memory difficulties" OR "visuospatial difficulties" OR "visuosensory difficulties" OR "visuomotor difficulties" OR "neuro* difficulties" OR "cognitive difficulties" OR "executive deficits" OR "executive difficulties" OR "self-regulation" OR "decision making" OR "reasoning" OR "planning" OR "flexibility" OR "dual-task*" OR "multi-task*" OR "problem solving" OR "spatial disorientation" OR "language deficits" OR "language difficulties" OR "language delay"<br>*Psychosocial level*<br>"psychosocial function*" OR "psychosocial difficulties" OR "psychological function* OR "psychological difficulties" OR "emotion* regulation" OR "emotion* difficulties" OR "distress" OR "stress" OR "quality of life" OR "self-esteem" OR "family stress" OR "family difficulties" OR "parental stress" OR "family difficulties" OR "sibling stress" OR "sibling difficulties" OR "burden of illness" OR "trauma" OR "psychopathology"

Notes. 1 = search terms including 'difficulties' were also replicated with 'problems' (e.g. "mood difficulties" OR "mood problems"). * = search terms accompanied by truncations to allow for orthographical variations in terminology. Disorder search term and behavioural search term sets were combined with 'AND'.
