# Supplementary material for: The research landscape of tuberous sclerosis complex–associated neuropsychiatric disorders (TAND)—a comprehensive scoping review
Source: J Neurodev Disord. 2022 Feb 13;14:13. doi: 10.1186/s11689-022-09423-3 (PMC8853020; doi:10.1186/s11689-022-09423-3)
Supplement: Supplementary file 2 — Additional file 2. Two hundred thirty studies included in scoping review according to study type. [file 11689_2022_9423_MOESM2_ESM.pdf]

Additional File 2. 230 studies included in scoping review according to study type

Animal Studies

1. Basu T, O'Riordan KJ, Schoenike BA, Khan NN, Wallace EP, Rodriguez G, et al. Histone deacetylase inhibitors restore normal hippocampal synaptic plasticity and seizure threshold in a mouse model of tuberous sclerosis complex. Sci Rep. 2019;9(1):5266.
2. Benthall KN, Cording KR, Agopyan-Miu AH, Chen EY, Bateup HS. Loss of TSC1 from striatal direct pathway neurons impairs endocannabinoid-LTD and enhances motor routine learning. bioRxiv. 2019;doi: https://doi.org/10.1101/2019.12.15.877126.
3. Cambiaghi M, Cursi M, Magri L, Castoldi V, Comi G, Minicucci F, et al. Behavioural and EEG effects of chronic rapamycin treatment in a mouse model of tuberous sclerosis complex. Neuropharmacol. 2013;67:1-7.
4. Carson RP, Fu C, Winzenburger P, Ess KC. Deletion of rictor in neural progenitor cells reveals contributions of mTORC2 signaling to tuberous sclerosis complex. Hum Mol Genet. 2013;22(1):140-152.
5. Chévere-Torres I, Maki JM, Santini E, Klann E. Impaired social interactions and motor learning skills in tuberous sclerosis complex model mice expressing a dominant/negative form of tuberin. Neurbiol Dis. 2012;45(1):156-164.
6. Ehninger D, Han S, Shilyansky C, Zhou Y, Li W, Kwiatkowski DJ, et al. Reversal of learning deficits in a $TSC2^{+/-}$ mouse model of tuberous sclerosis. Nat Med. 2008;14(8):843-848.
7. Ehninger D, Sano Y, de Vries PJ, Dies K, Franz D, Geschwind DH, et al. Gestational immune activation and TSC2 haploinsufficiency cooperate to disrupt fetal survival and may perturb social behavior in adult mice. Mol Psychiatry. 2012;17(1):62-70.
8. Ehninger D, Silva AJ. Increased levels of anxiety-related behaviors in a TSC2 dominant negative transgenic mouse model of tuberous sclerosis. Behav Genet. 2011;41(3):357-363.
9. Gkogkas CG, Khoutorsky A, Ran I, Rampakakis E, Nevarko T, Weatherill DB, et al. Autism-related deficits via dysregulated eIF4E-dependent translational control. Nature. 2013;493(7432):371-377.
10. Kelly E, Schaeffer SM, Dhamne SC, Lipton JO, Lindemann L, Honer M, et al. mGluR5 modulation of behavioral and epileptic phenotypes in a mouse model of tuberous sclerosis complex. Neuropsychopharmacol. 2018;43(6):1457-1465.

11. Kosillo P, Doig NM, Ahmed KM, Agopyan-Miu AH, Wong CD, Conyers L, et al. TSC1-mTORC1 signaling controls striatal dopamine release and cognitive flexibility. Nat Commun. 2019;10(1):5426.
12. McMahon JJ, Yu W, Yang J, Feng H, Helm M, McMahon E, et al. Seizure-dependent mTOR activation in 5-HT neurons promotes autism-like behaviors in mice. Neurobiol Dis. 2015;73:296-306.
13. Okamoto S, Prikhodko O, Pina-Crespo J, Adame A, McKercher SR, Brill LM, et al. Nitrosynapsin for the treatment of neurological manifestations of tuberous sclerosis complex in a rodent model. Neurobiol Dis. 2019;127:390-397.
14. Potter WB, Basu T, O'Riordan KJ, Kirchner A, Rutecki P, Burger C, et al. Reduced juvenile long-term depression in tuberous sclerosis complex is mitigated in adults by compensatory recruitment of mGluR5 and Erk signaling. PLoS Biol. 2013;11(8):e1001627.
15. Reith RM, McKenna J, Wu H, Hashmi SS, Cho S, Dash PK, Gambello MJ. Loss of TSC2 in purkinje cells is associated with autistic-like behavior in a mouse model of tuberous sclerosis complex. Neurobiol Dis. 2013;51:93-103.
16. Reith RM, Way S, McKenna III J, Haines K, Gambello MJ. Loss of the tuberous sclerosis complex protein tuberin causes purkinje cell degeneration. Neurobiol Dis. 2011;43(1):113-122.
17. Sato A, Kasai S, Kobayashi T, Takamatsu Y, Hino O, Ikeda K, et al. Rapamycin reverses impaired social interaction in mouse models of tuberous sclerosis complex. Nat Commun. 2012;3:1292.
18. Schneider M, de Vries PJ, Schönig K, Rößner V, Waltereit R. mTOR inhibitor reverses autistic-like social deficit behaviour in adult rats with both TSC2 haploinsufficiency and developmental status epilepticus. Eur Arch Psychiatry Clin Neurosci. 2017;267(5):455-463.
19. Tsai PT, Greene-Colozzi E, Goto J, Anderl S, Kwiatkowski DJ, Sahin M. Prenatal rapamycin results in early and late behavioral abnormalities in wildtype C57BI/6 mice. Behav Genet. 2013;43(1):51-59.
20. Tsai PT, Hull C, Chu Y, Greene-Colozzi E, Sadowski AR, Leech JM, et al. Autistic-like behaviour and cerebellar dysfunction in purkinje cell TSC1 mutant mice. Nature. 2012:488(7413):647-651.
21. Tsai PT, Rudolph S, Guo C, Ellegood J, Gibson JM, Schaeffer SM, et al. Sensitive periods for cerebellar-mediated autistic-like behaviors. Cell Rep. 2018;25(2):357-367.

22. von der Brelie C, Waltereit R, Zhang L, Beck H, Kirschstein T. Impaired synaptic plasticity in a rat model of tuberous sclerosis. Eur J Neurosci. 2006;23(3):686-692.
23. Waltereit R, Japs B, Schneider M, de Vries PJ, Bartsch D. Epilepsy and TSC2 haploinsufficiency lead to autistic-like social deficit behaviors in rats. Behav Genet. 2011;41(3):364-372.
24. Waltereit R, Welzl H, Dichgans J, Lipp H, Schmidt WJ, Weller M. Enhanced episodic-like memory and kindling epilepsy in a rat model of tuberous sclerosis. J Neurochem. 2006;96(2):407-413.
25. Way SW, Rozas NS, Wu HC, McKenna III J, Reith RM, Hashmi SS, et al. The differential effects of prenatal and/or postnatal rapamycin on neurodevelopmental defects and cognition in a neuroglial mouse model of tuberous sclerosis complex. Hum Mol Genet. 2012;21(14):3226-3236.
26. Young DM, Schenk AK, Yang S, Jan YN, Jan LY. Altered ultrasonic vocalizations in a tuberous sclerosis mouse model of autism. Proc Natl Acad Sci USA. 2010;107(24):11074-11079.
27. Yuan E, Tsai PT, Greene-Colozzi E, Sahin M, Kwiatkowski DJ, Malinowska IA. Graded loss of tuberin in an allelic series of brain models of TSC correlates with survival, and biochemical, histological and behavioral features. Hum Mol Genet. 2012;21(19):4286-4300.
28. Zeng L, Ouyang Y, Gazit V, Cirrito JR, Jansen LA, Ess KC, et al. Abnormal glutamate homeostasis and impaired synaptic plasticity and learning in a mouse model of tuberous sclerosis complex. Neurbiol Dis. 2007;28(2):184-196.
29. Zhang B, Guo D, Han L, Rensing N, Satoh A, Wong M. Hypothalamic orexin and mechanistic target of rapamycin activation mediate sleep dysfunction in a mouse model of tuberous sclerosis complex. Neurobiol Dis. 2020;134:104615.
30. Zhao J, Yoshii A. Hyperexcitability of the local circuit in mouse models of tuberous sclerosis complex. Mol Brain. 2019;12(1):6.

Case Studies

1. Al-Futaisi A, Idris A, Al-Sayegh A, Al-Mamari WS. Coexistence of autism spectrum disorders among three children with tuberous sclerosis complex: Case reports and review of literature. Sultan Qaboos Univ Med J. 2016;16(4):e520-e524.

2. Al-Sharbati M, Al-Zaidi R, Al-Naamani R, Al-Futaisi A, Jain R. A rare presentation of attention deficit/hyperactivity disorder: A recommendation to be more alert! Sultan Qaboos Univ Med J. 2010;10(1):84-88.
3. Bhattacharya A, Das S, Nath K, Dutta D, Saddichha S. Atypical presentation of tuberous sclerosis and obsessive compulsive disorder in an adult male. Ann Indian Acad Neurol. 2012;15(2):161-162.
4. Boronat S, van Eeghen AM, Shinnick JE, Newberry P, Thiele EA. Stressor-related disorders in tuberous sclerosis. Ann Clin Psychiatry. 2013;25(4):243-249.
5. Butt DS, Kamran M, Ussaid A, Ullah Z, Butt KI. Tuberous sclerosis complex: Case report and management by multidisciplinary approach. J Pak Assoc Dermatol. 2017;27(3):301-305.
6. Chopra VK, Cintury Y, Sinha VK. Bipolar disorder associated with tuberous sclerosis: Chance association or aetiological relationship? Indian J Psychiatry. 2006;48(1):66-68.
7. Coelho MP, Klumpp M, Majmudar A, Gupta S. Incidental finding of renal pseudoaneurysm in child with tuberous sclerosis presenting with atypical clinical symptoms. Radiol Case Rep. 2019;14(10):1193-1196.
8. Coppola G, Klepper J, Ammendola E, Fiorillo M, della Corte R, Capano G, et al. The effects of the ketogenic diet in refractory partial seizures with reference to tuberous sclerosis. Eur J Paediatr Neurol. 2006;10(3):148-151.
9. Datta AK, Mandal S, Bhattacharya S. Autism and mental retardation with convulsion in tuberous sclerosis: A case report. Cases J. 2009;2:7061.
10. Fryer AE, Osborne JP, Tan R, Siggers DC. Tuberous sclerosis: A large family with no history of seizures or mental retardation. J Med Genet. 1987;24:547-548.
11. Ghosh S, Chaudhury PK, Bhattacharya A, Praharaj SK. Tuberous sclerosis presenting as psychosis in an adolescent patient. J Neuropsychiatry Clin Neurosci. 2014;26(1):E38.
12. Gipson TT, Jennett H, Wachtel L, Gregory M, Poretti A, Johnston MV. Everolimus and intensive behavioral therapy in an adolescent with tuberous sclerosis complex and severe behavior. Epilepsy Behav Case Rep. 2013;1:122-125.
13. Gipson TT, Poretti AP. Implementing a multidisciplinary approach to treating tuberous sclerosis complex: A case report. Child Neurol Open. 2017;4:2329048X17725609.
14. Gipson TT, Poretti A, Thomas EA, Jenkins KT, Desai S, Johnston MV. Autism phenotypes in tuberous sclerosis complex: Diagnostic and treatment considerations. J Child Neurol. 2015;30(14):1871-1876.

15. Glavan N, Ljubičić-Bistrović I, Grahovac B, Traven L, Sasso A, Jonjić N. Ungual fibroma in 12-year-old boy with hypomelanotic macules, intellectual disability and attention deficit hyperactivity disorder–possible tuberous sclerosis. SAGE Open Med Case Rep. 2016;26;4:2050313X16666233.
16. Goh S, Thiele EA. Anorexia nervosa in a child with tuberous sclerosis complex. J Child Neurol. 2005;20(5):457-560.
17. Hassan IK, Looi JC, Velakoulis D, Gaillard F, Lui EH, O'Brien TJ, et al. Psychosis with obsessive-compulsive symptoms in tuberous sclerosis. J Clin Neurosci. 2014;21(5):867-869.
18. Humphrey A, Higgins JN, Yates JR, Bolton PF. Monozygotic twins with tuberous sclerosis discordant for the severity of developmental deficits. Neurology. 2004;62(5):795-798.
19. Humphrey A, Neville BG, Clarke A, Bolton PF. Autistic regression associated with seizure onset in an infant with tuberous sclerosis. Dev Med Child Neurol. 2006;48(7):609-611.
20. Hwang S, Lee J, Yang J, Lim C, Lee J, Lee Y, et al. Everolimus improves neuropsychiatric symptoms in a patient with tuberous sclerosis carrying a novel TSC2 mutation. Mol Brain. 2016;9(1):56.
21. Ishii R, Wataya-Kaneda M, Canuet L, Nonomura N, Nakai Y, Takeda M. Everolimus improves behavioral deficits in a patient with autism associated with tuberous sclerosis: A case report. Neuropsychiatr Electrophysiol. 2015;1:6.
22. Khanna R, Borde M. Mania in a five-year-old child with tuberous sclerosis. Br J Psychiatry. 1989;155:117-119.
23. Kilincaslan A, Kok BE, Tekturk P, Yalcinkaya C, Ozkara C, Yapici Z. Beneficial effects of everolimus on autism and attention-deficit-hyperactivity disorder symptoms in a group of patients with tuberous sclerosis complex. J Child Adolesc Psychopharmacol. 2017;27(4):383-388.
24. Konda S. Tuberous sclerosis presenting with confusion and agitation. Clin Med (Lond). 2011;11(5):502-503.
25. Kossoff EH, Thiele EA, Pfeifer HH, McGrogan JR, Freeman JM. Tuberous sclerosis complex and the ketogenic diet. Epilepsia. 2005;46(10):1684-1686.
26. Kothur K, Ray M, Malhi P. Correlation of autism with temporal tubers in tuberous sclerosis complex. Neurol India. 2008;56(1):74-76.

27. Lawlor BA, Maurer RG. Tuberous sclerosis and the autistic syndrome. Br J Psychiatry. 1987;150:396-397.
28. Lee S, Min J, Lee I, Kim JJ. Clinical usefulness of aripiprazole and lamotgrigine in schizoaffective presentation of tuberous sclerosis. Clin Psychopharmacol Neurosci. 2016;14(3):305-310.
29. Liu Q, Gao J. Psychiatric symptoms in an individual with tuberous sclerosis. Shanghai Arch Psychiatry. 2012;24(4):233-234.
30. Marinelli T, Chen L, Kulkarni J. First episode psychosis in an adult patient with tuberous sclerosis. Aust N Z J Psychiatry. 2016;50(3):292-293.
31. Oliver BE. Tuberous sclerosis and the autistic syndrome. Br J Psychiatry. 1987;151:560.
32. Ozgur BG, Aksu H, Tosun AF. Comorbid obsessive compulsive disorder in a child with tuberous sclerosis complex. PBS 2018;8(3):142-144.
33. Rao SA, Ra MG, Rao NP, Varambally S, Gangadhar BN. Successful treatment of tuberous sclerosis with psychosis and obsessive-compulsive disorder: A case report. Psychiatry Clin Neurosci. 2015;69(8):504-505.
34. Reich M, Lenoir P, Malvy J, Perrot A, Sauvage D. Sclérose tubéreuse de Bourneville et autism. Arch Pédiatr. 1997;4:170-175.
35. Rezaie R, Zouridakis G, Choudhri AS, Wheless JW, Völgyi E, Van Poppel K, et al. Functional connectivity in tuberous sclerosis complex with autistic spectrum disorder preliminary findings. J Pediatr Neurol. 2013;11(2):79-82.
36. Samanta D. Remarkable improvement of selective mutism with everolimus in a patient with tuberous sclerosis complex. Acta Neurol Bel. 2015;115(4):815-817.
37. Sedky K, Hughes T, Yusufzie K, Lippmann S. Tuberous sclerosis with psychosis. Psychosomatics. 2003;44(6):521-522.
38. Sharma P, Rao K. Psychological intervention in tuberous sclerosis: A case report. Indian J Psychiatry. 2002;44(4):391-396.
39. Sitholey P, Aga VM, Agarwal V, Prasad M. Childhood autism in tuberous sclerosis. Indian J Pediatr. 1998;65:615-617.
40. Takanashi J, Sugita K, Fujii K, Niimi H. MR evaluation of tuberous sclerosis: Increased sensitivity with fluid-attenuated inversion recovery and relation to severity of seizures and mental retardation. AJNR Am J Neuroradiol. 1995;16(9):1923-1928.
41. Tasdemir A, Kalelioglu T, Genc A, Kalelioglu T, Umut G. Psychosis in a female patient with tuberous sclerosis. J Neuropsychiatry Clin Neurosci. 2013;25(2):E61.

42. Timotin L, Sarrot-Reynauld F, Lantuejoul S, Pasquier B, Massot C, Ashraf A, et al. Sclérose tubéreuse de Bourneville sans altération intellectuelle, diagnostiquée à l'âge adulte. Rev Med Interne. 2005;26(6):511-513.
43. Tye C, Varcin K, Bolton P, Jeste SS. Early developmental pathways to autism spectrum disorder in tuberous sclerosis complex. Adv Autism. 2016;2(2):84-93.
44. Vlaskamp C, Poil S, Jansen F, Linkenkaer-Hansen K, Durston S, Oranje B, et al. Bumetanide as a candidate treatment for behavioral problems in tuberous sclerosis complex. Front Neurol. 2017;8:469.
45. Waszak PM, Lewandowska K, Kasprzycka-Waszak W, Gordon W, Zagozdzon P. Tuberous sclerosis-associated neuropsychiatric disorders–case report. Neuropsychiatr I Neuropsychol. 2017;12(2):73-79.
46. Willacy H. The impact of tuberous sclerosis complex–a parent's perspective. Adv Autism. 2016;2(2):70-75.
47. Yui K, Imataka G, Sasaki H, Kawasaki Y, Okanshi T, Shiroki R, et al. Improvement in impaired social cognition but not seizures by everolimus in a child with tuberous sclerosis-associated autism through increased serum antioxidant proteins and oxidant/antioxidant status. Case Rep Pediatr. 2019;1-10.

Cohort Studies

1. Adams D, Hastings RP, Alston-Knox C, Cianfiaglione R, Eden K, Felce D, et al. Using Bayesian methodology to explore the profile of mental health and well-being in 646 mothers of children with 13 rare genetic syndromes in relation to mothers of children with autism. Orphanet J Rare Dis. 2018;13(1):185.
2. Amin S, Mallick AA, Lux A, O'Callaghan F. Quality of life in patients with tuberous sclerosis complex (TSC). Eur J Paediatr Neurol. 2019;23(6):801-807.
3. Asano E, Chugani DC, Muzik O, Behen M, Janisse J, Rothermel R, et al. Autism in tuberous sclerosis complex is related to both cortical and subcortical dysfunction. Neurology. 2001;57(7):1269-1277.
4. Baker P, Piven J, Sato Y. Autism and tuberous sclerosis complex: Prevalence and clinical features. J Autism Dev Disord. 1998;28(4):279-285.
5. Bar C, Ghobeira R, Azzi R, Ville D, Riquet A, Touraine R, et al. Experience of follow-up, quality of life, and transition from pediatric to adult healthcare of patients with tuberous sclerosis complex. Epilepsy Behav. 2019;96:23-27.

6. Baumer FM, Peters JM, Clancy S, Prohl AK, Prabhu SP, Scherrer B, et al. Corpus callosum white matter diffusivity reflects cumulative neurological comorbidity in tuberous sclerosis complex. Cereb Cortex. 2018;28(10):3665-3672.
7. Benova B, Petrak B, Kyncl M, Jezdik P, Maulisova A, Jahodova A, et al. Early predictors of clinical and mental outcome in tuberous sclerosis complex: A prospective study. Eur J Paediatr Neurol. 2018;22(4):632-641.
8. Bolton PF, Clifford M, Tye C, Maclean C, Humphrey A, le Maréchal K, et al. Intellectual abilities in tuberous sclerosis complex: Risk factors and correlates from the tuberous sclerosis 2000 study. Psychol Med. 2015;45(11):2321-2331.
9. Bolton PF, Griffiths PD. Association of tuberous sclerosis of temporal lobes with autism and atypical autism. Lancet. 1997;349:392-395.
10. Bolton PF, Park RJ, Higgins NP, Griffiths PD, Pickles A. Neuro-epileptic determinants of autism spectrum disorders in tuberous sclerosis complex. Brain, 2002;125(6):1247-1255.
11. Bombardieri R, Pinci M, Moavero R, Cerminara C, Curatolo P. Early control of seizures improves long-term outcome in children with tuberous sclerosis complex. Eur J Paediatr Neurol. 2010;14(2):146-149.
12. Both P, ten Holt L, Mous S, Patist J, Rietman A, Dieleman G, et al. Tuberous sclerosis complex: Concerns and needs of patients and parents from the transitional period to adulthood. Epilepsy Behav. 2018;83:13-21.
13. Bruni O, Cortesi F, Giannotti F, Curatolo P. Sleep disorders in tuberous sclerosis: A polysomnographic study. Brain Dev. 1995;17(1):52-56.
14. Capal JK, Bernardino-Cuesta B, Horn PS, Murray D, Byars AW, Bing NM, et al. Influence of seizures on early development in tuberous sclerosis complex. Epilepsy Behav. 2017;70:245-252.
15. Capal JK, Horn PS, Murray DS, Byars AW, Bing NM, Kent B, et al. Utility of the Autism Observation Scale for Infants in early identification of autism in tuberous sclerosis complex. Pediatr Neurol. 2017;75:50-86.
16. Chou I, Lin K, Wong AM, Wang H, Chou M, Hung P, et al. Neuroimaging correlation with neurological severity in tuberous sclerosis complex. Eur J Paediatr Neurol. 2018;12(2):108-112.
17. Chung CW, Lawson JA, Sarkozy V, Riney K, Wargon O, Shand AW, et al. Early detection of tuberous sclerosis complex: an opportunity for improved neurodevelopmental outcome. Pediatr Neurol. 2017;76:20-26.

18. Chung TK, Lynch ER, Fiser CJ, Nelson DA, Agricola K, Tudor C, et al. Psychiatric comorbidity and treatment response in patients with tuberous sclerosis complex. Ann Clin Psychiatry. 2011;23(4):263-269.
19. Chu-Shore CJ, Major P, Camposano S, Muzykewicz D, Thiele EA. The natural history of epilepsy in tuberous sclerosis complex. Epilepsia. 2010;51(7):1236-1241.
20. Cook IA, Wilson AC, Peters JM, Goyal MN, Bebin EM, Northrup H, et al. EEG spectral features in sleep of autism spectrum disorders in children with tuberous sclerosis complex. J Autism Dev Disord. 2020;50(3):916-923.
21. Crall C, Valle M, Kapur K, Dies KA, Liang MG, Sahin M, et al. Effect of angiofibromas on quality of life and access to care in tuberous sclerosis patients and their caregivers. Pediatr Dermatol. 2016;33(5):518-525.
22. Curatolo P, Moavero R. Autism in tuberous sclerosis: are risk factors identifiable and preventable? Future Neurol. 2011;6(4):451-454.
23. Cusmai R, Moavero R, Bombardieri R, Vigevano F, Curatolo P. Long-term neurological outcome in children with early-onset epilepsy associated with tuberous sclerosis. Epilepsy Behav. 2011;22(4):735-739.
24. de Vries PJ, Belousava E, Benedik MJ, Carter T, Cottin V, Curatolo P, et al. TSC-associated neuropsychiatric disorders (TAND): Findings from the TOSCA natural history study. Orphanet J Rare Dis. 2018;13(1):157.
25. de Vries PJ, Franz DN, Curatolo P, Nabbout R, Neary M, Herbst F, et al. Measuring health-related quality of life in tuberous sclerosis complex–psychometric evaluation of three instruments in individuals with refractory epilepsy. Front Pharmacol. 2018;9:964.
26. de Vries PJ, Gardiner J, Bolton PF. Neuropsychological attention deficits in tuberous sclerosis complex (TSC). Am J Med Genet. 2009;149A(3):387-395.
27. de Vries PJ, Hunt A, Bolton PF. The psychopathologies of children and adolescents with tuberous sclerosis complex (TSC): A postal survey of UK families. Eur Child Adolesc Psychiatry. 2007;16(1):16-24.
28. de Vries PJ, Watson P. J. Attention deficits in tuberous sclerosis complex (TSC): rethinking the pathway to the endstate. Intellect Disabil Res. 2008;52(4):348-357.
29. Dickinson A, Varcin KJ, Sahin M, Nelson III, CA, Jeste SS. Early patterns of functional brain development associated with autism spectrum disorder in tuberous sclerosis complex. Autism Res. 2019;12(12):1758-1773.
30. Ebrahimi-Fakhari D, Hussong J, Flotats-Bastardas M, Ebrahimi-Fakhari D, Zemlin M, von Gontard A, et al. Tuberous sclerosis complex associated neuropsychiatric disorders

and parental stress: Findings from a national, prospective TSC surveillance study. Neuropediatrics. 2019;50(5):294-299.

31. Eden KE, de Vries PJ, Moss J, Richards C, Oliver C. Self-injury and aggression in tuberous sclerosis complex: Cross syndrome comparison and associated risk markers. J Neurodev Disord. 2014;6(1):10.
32. Eluvathingal TJ, Behen ME, Chugani HT, Janisse J, Bernardi B, Chakraborty P, et al. Cerebellar lesions in tuberous sclerosis complex: Neurobehavioral and neuroimaging correlates. J Child Neurol. 2006;21(10):846-851.
33. Farach LS, Pearson DA, Woodhouse JP, Schraw JM, Sahin M, Krueger DA, et al. Tuberous sclerosis complex genotypes and developmental phenotype. Pediatr Neurol. 2019;96:58-63.
34. Ferguson AP, McKinlay IA, Hunt A. Care of adolescents with severe learning disability from tuberous sclerosis. Dev Med Child Neurol. 2002;44:256-262.
35. Fong CY, Ng K, Kong AN, Ong LC, Rithauddin MA, Thong MK, et al. Quality of life of children with tuberous sclerosis complex. Arch Dis Child. 2019;104(10):972-978.
36. Gallagher A, Tanaka N, Suzuki N, Liu H, Thiele EA, Stufflebeam SM. Decreased language laterality in tuberous sclerosis complex: A relationship between language dominance and tuber location as well as history of epilepsy. Epilepsy Behav. 2012;25(1):36-41.
37. Gallagher A, Tanaka N, Suzuki N, Liu H, Thiele EA, Stufflebeam SM. Diffuse cerebral language representation in tuberous sclerosis complex. Epilepsy Res. 2013;104(1-2):125-133.
38. Gillberg JC, Gillberg C, Ahlsen G. Autistic behaviour and attention deficits in tuberous sclerosis: a population-based study. Dev Med Child Neurol. 1994:36:50-56.
39. Gipson TT, Poretti A, Kelley SA, Carson KA, Johnston MV, Huisman TA. Characterization of the basal ganglia using diffusion tensor imaging in children with self-injurious behavior and tuberous sclerosis complex. J Neuroimaging. 2019;29(4):506-511.
40. Goh S, Kwiatkowksi DJ, Dorer DJ, Thiele EA. Infantile spasms and intellectual outcomes in children with tuberous sclerosis complex. Neurology. 2005;65(2):235-238.
41. Graffigna G, Bosio C, Cecchini I. Assisting a child with tuberous sclerosis complex (TSC): A qualitative deep analysis of parents' experience and caring needs. BMJ Open. 2013;3:e003707.

42. Granader YE, Bender HA, Zemon V, Rathi S, Nass R, MacAllister WS. The clinical utility of the social responsiveness scale and social communication questionnaire in tuberous sclerosis complex. Epilepsy Behav. 2010;18(3):262-266.
43. Guo X, Tu W, Shi X. Tuberous sclerosis complex in autism. Iran J Pediatr. 2012;22(3):408-411.
44. Gutierrez G, Smalley SL, Tanguay PE. Autism in tuberous sclerosis complex. J Autism Dev Disord. 1998;28(2):97-103.
45. Hancock E, O'Callaghan F, English J, Osborne JP. Melatonin excretion in normal children and in tuberous sclerosis complex with sleep disorder responsive to melatonin. J Child Neurol. 2005;20(1):21-25.
46. Hancock E, O'Callaghan F, Osborne J. Effect of melatonin dosage on sleep disorder in tuberous sclerosis complex. J Child Neurol. 2005;20(1):78-80.
47. Harrison JE, O'Callaghan FJ, Hancock E, Osborne JP, Bolton PF. Cognitive deficits in normally intelligent patients with tuberous sclerosis. Am J Med Genet. 1999;88:642-646.
48. Huang C, Peng SS, Weng W, Su Y, Lee W, National Taiwan University Hospital Tuberous Sclerosis Complex Study Group. The relationship of neuroimaging findings and neuropsychiatric comorbidities in children with tuberous sclerosis complex. J Formos Med Assoc. 2015;114(9):849-854.
49. Humphrey A, MacLean C, Ploubidis GB, Granader Y, Clifford M, Haslop M, et al. Intellectual development before and after the onset of infantile spasms: A controlled prospective longitudinal study in tuberous sclerosis. Epilepsia. 2014;55(1):108-116.
50. Humphrey A, Williams J, Pinto E, Bolton PF. A prospective longitudinal study of early cognitive development in tuberous sclerosis: A clinic based study. Eur Child Adolesc Psychiatry. 2004;13(3):159-165.
51. Hunt A. Development, behaviour and seizures in 300 cases of tuberous sclerosis. J Intellect Disabil Res. 1993;37:41-51.
52. Hunt A. A comparison of the abilities, health and behaviour of 23 people with tuberous sclerosis at age 5 and as adults. J Appl Res Intellect Disabil. 1998;11(3):227-238.
53. Hunt A, Stores G. Sleep disorder and epilepsy in children with tuberous sclerosis: a questionnaire-based study. Dev Med Child Neurol. 1994;36(2):108-115.
54. Ierardi AM, Petrillo M, Coppola A, Angileri SA, Galassi A, Padovano B, et al. Percutaneous microwave ablation or renal angiomyolipomas in tuberous sclerosis complex to improve the quality of life: Preliminary experience in an Italian center. Radiol Med. 2019;124(3):176-183.

55. Im K, Ahtam B, Haehn D, Peters JM, Warfield SK, Sahin M, et al. Altered structural brain networks in tuberous sclerosis complex. Cereb Cortex. 2016;26(5):2046-2058.
56. Jambaqué I, Chiron C, Dumas C, Mumford J, Dulac O. Mental and behavioural outcome of infantile epilepsy treated by vigabatrin in tuberous sclerosis patients. Epilepsy Res. 2000;38(2-3):151-160.
57. Jansen FF, Braams O, Vincken KL, Algra A, Anbeek P, Jennekens-Schinkel A, et al. Overlapping neurologic and cognitive phenotypes in patients with TSC1 or TSC2 mutations. Neurology. 2008;70(12):908-915.
58. Jansen FE, Vincken KL, Algra A, Anbeek P, Braams O, Nellist M, et al. Cognitive impairment in tuberous sclerosis complex is a multifactorial condition. Neurology. 2008;70(12):916-923.
59. Jeste SS, Hirsch S, Vogel-Farley V, Norona A, Navalta M, Gregas MC, et al. Atypical face processing in children with tuberous sclerosis complex. J Child Neurol. 2012;28(12):1569-1576.
60. Jeste SS, Sahin M, Bolton P, Ploubidis GB, Humphrey A. Characterization of autism in young children with tuberous sclerosis complex. J Child Neurol. 2008;23(5):520-525.
61. Jeste SS, Varcin KJ, Hellemann GS, Gulsrud AC, Bhatt R, Kasari C, et al. Symptom profiles of autism spectrum disorder in tuberosis sclerosis complex. Neurology. 2016;87(8):766-772.
62. Jeste SS, Wu JY, Senturk D, Varcin K, Ko J, McCarthy B, et al. Early developmental trajectories associated with ASD in infants with tuberous sclerosis complex. Neurology. 2014;83(2):160-168.
63. Joinson C, O'Callaghan FJ, Osborne JP, Martyn C, Harris T, Bolton PF. Learning disability and epilepsy in an epidemiological sample of individuals with tuberous sclerosis complex. Psychol Med. 2003;33(2):335-344.
64. Jozwiak S, Goodman M, Lamm SH. Poor mental development in patients with tuberous sclerosis complex. Arch Neurol. 1998;55(3):379-384.
65. Jozwiak S, Kotulska K, Domanska-Pakiela D, Lojszczyk B, Syczewska M, Chmielewski D, et al. Antiepileptic treatment before the onset of seizures reduces epilepsy severity and risk of mental retardation in infants with tuberous sclerosis complex. Eur J Paediatr Neurol. 2011;15(5):424-431.
66. Kaczorowska M, Jurkiewicz E, Domanska-Pakiela D, Syczewska M, Lojszczyk B, Chmielewski D, et al. Cerebral tuber count and its impact on mental outcome of patients with tuberous sclerosis complex. Epilepsia. 2011;52(1):22-27.

67. Kassiri J, Snyder TJ, Bhargava R, Wheatly BM, Sinclair DB. Cortical tubers, cognition, and epilepsy in tuberous sclerosis. Pediatr Neurol. 2011;44(5):328-332.
68. Kingswood C, Bolton P, Crawford P, Harland C, Johnson SR, Sampson JR, et al. The clinical profile of tuberous sclerosis complex (TSC) in the United Kingdom: A retrospective cohort study in the Clinical Practice Research Datalink (CPRD). Eur J Paediatr Neurol. 2016;20(2):296-308.
69. Kingswood JC, d'Augères GB, Belousova E, Ferreira JC, Carter T, Castellana R, et al. TuberOus SClerosis registry to increase disease awareness (TOSCA)–baseline data on 2093 patients. Orphanet J Rare Dis. 2017;12(1):2.
70. Kopp CM, Muzykewicz DA, Staley BA, Thiele EA, Pulsifer MB. Behavior problems in children with tuberous sclerosis complex and parental stress. Epilepsy Behav. 2008;13(3):505-510.
71. Kothare SV, Singh K, Hochman T, Chalifoux JR, Staley BA, Weiner HL, et al. Genotype/phenotype in tuberous sclerosis complex: Associations with clinical and radiologic manifestations. Epilepsia. 2014;55(7):1020-1024.
72. Krueger DA, Sadhwani A, Byars AW, de vries PJ, Franz DN, Whittemore VH, et al. Everolimus for treatment of tuberous sclerosis complex-associated neuropsychiatric disorders. Ann Clin Transl Neurol. 2017;4(12):877-887.
73. Krueger DA, Wilfong AA, Holland-Bouley KH, Anderson AE, Agricola K, Tudor C, et al. Everolimus treatment of refractory epilepsy in tuberous sclerosis complex. Ann Neurol. 2013;74(5):679-687.
74. Leclezio L, Gardner-Lubbe S, de Vries PJ. Is it feasible to identify natural clusters of TSC-Associated Neuropsychiatric Disorders (TAND)? Pediatr Neurol. 2018;81:38-44.
75. Lennert B, Farrelly E, Sacco P, Pira G, Frost M. Resource utilization in children with tuberous sclerosis complex and associated seizures: A retrospective chart review study. J Child Neurol. 2013;28(4):461-469.
76. Lewis WW, Sahin M, Scherrer B, Peters JM, Suarez RO, Vogel-Farley VK, et al. Impaired language pathways in tuberous sclerosis complex patients with autism spectrum disorders. Cereb Cortex. 2013;23(7):1526-1532.
77. Lewis JC, Thomas HV, Murphy KC, Sampson JR. Genotype and psychological phenotype in tuberous sclerosis. J Med Genet. 2004;41:203-207.
78. Liang S, Li A, Zhao M, Jiang H, Yu S, Meng X, et al. Epilepsy surgery in tuberous sclerosis complex: Emphasis on surgical candidate and neuropsychology. Epilepsia. 2010;51(11):2316-2321.

79. Lyczkowski DA, Conant KD, Pulsifer MB, Jarrett DY, Grant PE, Kwiatkowksi DJ, et al. Intrafamilial phenotypic variability in tuberous sclerosis complex. J Child Neurol. 2007;22(12):1348-1355.
80. Marques R, Belousova E, Benedik MP, Carter T, Cottin V, Curatolo P, et al. Treatment patterns and use of resources in patients with tuberous sclerosis complex: Insights from the TOSCA Registry. Fron Neurol. 2019;10:1144.
81. Matsuyama K, Ohsawa I, Ogawa T. Do children with tuberous sclerosis complex have superior musical skill?–A unique tendency of musical responsiveness in children with TSC. Med Sci Monit. 2007;13(4):CR156-164.
82. McDonald A, Goodwin J, Roberts S, Fish L, Vaughan B, Cooper A, et al. 'We've made the best of it. But we do not have a normal life': Families' experiences of tuberous sclerosis complex and seizure management. J Intellect Disabil Res. 2019;63(8):947-956.
83. McDonald NM, Varcin KJ, Bhatt R, Wu JY, Sahin M, Nelson III CA, et al. Early autism symptoms in infants with tuberous sclerosis complex. Autism Res. 2017;10(12):1981-1990.
84. Mizuguchi M, Ikeda H, Kagitani-Shimono K, Yoshinaga H, Suzuki Y, Aoki M, et al. Everolimus for epilepsy and autism spectrum disorder in tuberous sclerosis complex: EXIST-3 substudy in Japan. Brain Dev. 2019;41(1):1-10.
85. Moavero R, Benvenuto A, Gialloreti LE, Siracusano M, Kotulska K, Weschke B, et al. Early clinical predictors of autism spectrum disorder in infants with tuberous sclerosis complex: Results from the EPISTOP Study. J Clin Med. 2019;8(6):788.
86. Moavero R, Napolitano A, Cusmai F, Vigevano F, Figà-Talamanca L, Calbi G, et al. White matter disruption is associated with persistent seizures in tuberous sclerosis complex. Epilepsy Behav. 2016;60:63-67.
87. Monteiro T, Garrido C, Pina S, Chorão R, Carrilho I, Figueiroa S, et al. Tuberous sclerosis: Clinical characteristics and their relationship to genotype/phenotype. An Pediatr. 2014;81(5):289-296.
88. Morrison PJ, O'Neill T, Hardy R, Shepherd CW, Donnelly DE. The prevalence of pica in tuberous sclerosis complex. Springerplus. 2015;4:51.
89. Mous SE, Overwater IR, Gato RV, Duvekot J, ten Hoopen LW, Lequin MH, et al. Cortical dysplasia and autistic trait severity in children with tuberous sclerosis complex: A clinical epidemiological study. Eur Child Adolesc Psychiatry. 2018;27(6):753-765.

90. Mowrey KE, Ashfaq M, Pearson DA, Hashmi SS, Roberds SL, Farach LS, et al. The impact of psychiatric symptoms on tuberous sclerosis complex and utilization of mental health treatment. Pediatr Neurol. 2019;91:41-49.
91. Muzykewicz DA, Newberry P, Danforth N, Halpern EF, Thiele EA. Psychiatric comorbid conditions in a clinic population of 241 patients with tuberous sclerosis complex. Epilepsy Behav. 2007;11(4):506-513.
92. Numis AL, Major P, Monenegro MA, Muzykewicz DA, Pulsifer MB, Thiele EA. Identification of risk factors for autism spectrum disorders in tuberous sclerosis complex. Neurology. 2011;76(11):981-987.
93. O'Callaghan FJ, Clarke AA, Hancock E, Hunt A, Osborne JP. Use of melatonin to treat sleep disorders in tuberous sclerosis. Dev Med Child Neurol. 1999;41(2):123-126.
94. O'Callaghan FJ, Harris T, Joinson C, Bolton P, Noakes M, Presdee D, et al. The relation of infantile spasms, tubers, and intelligence in tuberous sclerosis complex. Arch Dis Child. 2004;89(6):530-533.
95. Ochi A, Hung R, Weiss S, Widjaja E, To T, Nawa Y, et al. Lateralized interictal epileptiform discharge during rapid eye movement sleep correlate with epileptogenic hemisphere in children with intractable epilepsy secondary to tuberous sclerosis complex. Epilepsia. 2011;52(11):1986-1994.
96. Overwater IE, Rietman AB, Mous, SE, Bindels-de Heus K, Rizopoulos D, ten Hoopen LW, et al. A randomized controlled trial with everolimus for IQ and autism in tuberous sclerosis complex. Neurology. 2019;93(2):e200-e209.
97. Overwater IE, Verhaar BJ, Lingsma HF, Bindels-de Heus GC, van den Ouweland AM, Nellist M, et al. Interdependence of clinical factors predicting cognition in children with tuberous sclerosis complex. J Neurol. 2017;264(1):161-167.
98. Paprocka J, Kijonka M, Boguszewicz L, Sokól M. Melatonin in tuberous sclerosis complex analysis using modern mathematical modeling methods. Int J Endocrinol. 2017;2017:8234502.
99. Park RJ, Bolton PF. Pervasive developmental disorder and obstetric complications in children and adolescents with tuberous sclerosis. Autism. 2001;5(3):237-248.
100. Park S, Lee EJ, Eom S, Kang H, Lee JS, Kim HD. Ketogenic diet for the management of epilepsy associated with tuberous sclerosis complex in children. J Epilepsy Res. 2017;7(1):45-49.
101. Parker M. Families caring for chronically ill children with tuberous sclerosis complex. Fam Community Health. 1996;19(3):73-84.

102. Pascual-Castroviejo I, Hernández-Moneo JL, Pascual-Pascual SI, Viaño J, Gutiérrez-Molina M, Velazquez-Fragua R, et al. Significance of tuber size for complications of tuberous sclerosis complex. Neurología. 2013;28(9):550-557.

103. Peters JM, Taquet M, Vega C, Jeste SS, Fernandez IS, Tan J, et al. Brain functional networks in syndromic and non-syndromic autism: A graph theoretical study of EEG connectivity. BMC Med. 2013;11(1):54.

104. Prohl AK, Scherrer B, Tomas-Fernandez X, Davis PE, Filip-Dhima R, Prabhu SP, et al. Early white matter development is abnormal in tuberous sclerosis complex patients who develop autism spectrum disorder. J Neurodev Disord. 2019;11(1):36.

105. Pulsifer MB, Winterkorn EB, Thiele EA. Psychological profile of adults with tuberous sclerosis complex. Epilepsy Behav. 2007;10(3):402-406.

106. Raznahan A, Higgins NP, Griffiths PD, Humphrey A, Yates JR, Bolton PF. Biological markers of intellectual disability in tuberous sclerosis. Psychol Med. 2007;37(9):1293-304.

107. Raznahan A, Joinson C, O'Callaghan F, Osborne JP, Bolton PF. Psychopathology in tuberous sclerosis: an overview and findings in a population-based sample of adults with tuberous sclerosis. J Intellect Disabil Res. 2006;50(8):561-569.

108. Rentz AM, Skalicky AM, Liu Z, Dunn DW, Frost MD, Nakagawa JA, et al. Burden of renal angiomyolipomas associated with tuberous sclerosis complex: Results of a patient and caregiver study. J Patient Rep Outcomes. 2018;2(1):30.

109. Rentz AM, Skalicky AM, Liu Z, Wheless JW, Dunn DW, Frost MD, et al. Tuberous sclerosis complex: a survey of health care resource use and health burden. Pediatr Neurol. 2015;52(4):435-441.

110. Rentz AM, Skalicky AM, Pashos CL, Liu Z, Magestro M, Pelletier CL, et al. Caring for children with tuberous sclerosis complex: What is the physical and mental health impact on caregivers? J Child Neurol. 2015;30(12):1574-1581.

111. Ridler K, Suckling J, Higgins NJ, de Vries PJ, Stephenson CM, Bolton PF, et al. Neuroanatomical correlates of memory deficits in tuberous sclerosis complex. Cereb Cortex. 2007;17(2):261-271.

112. Roth J, Olasunkanmi A, MacAllister WS, Weil E, Uy CC, Devinsky O, et al. Quality of life following epilepsy surgery for children with tuberous sclerosis complex. Epilepsy Behav. 2011;20(3):561-565.

113. Samia P, Donald KA, Schlegel B, Wilmshurst JM. Parental understanding of tuberous sclerosis complex. J Child Neurol. 2015;30(10):1281-1286.

114. Samir H, Ghaffar HA, Nasr M. Seizures and intellectual outcome: Clinico-radiological study of 30 Egyptian cases of tuberous sclerosis complex. Eur J Paediatr Neurol. 2011;15(2):131-137.
115. Scherrer B, Prohl AK, Taquet M, Kapur K, Peters JM, Tomas-Fernandez X, et al. The connectivity fingerprint of the fusiform gyrus captures the risk of developing autism in infants with tuberous sclerosis complex. Cereb Cortex. 2020;30(4):2199-2214.
116. Schoenberger A, Capal JK, Ondracek A, Horn PS, Murray D, Byars AW, et al. Language predictors of autism spectrum disorder in young children with tuberous sclerosis complex. Epilepsy Behav. 2020;103:106844.
117. Seri S, Cerquiglini A, Pisani F, Curatolo P. Autism in tuberous sclerosis: Evoked potential evidence for a deficit in auditory sensory processing. Clin Neurophysiol. 1999;110:1825-1830.
118. Shepherd C, Koepp M, Myland M, Patel K, Miglio C, Siva V, et al. Understanding the health economic burden of patients with tuberous sclerosis complex (TSC) with epilepsy: A retrospective cohort study in the UK Clinical Practice Research Datalink (CPRD). BMJ Open. 2017;7(10):e015236.
119. Shepherd CW, Houser OW, Gomez MR. MR findings in tuberous sclerosis complex and correlation with seizure development and mental impairment. AJNR Am J Neuroradiol. 1995;16(1):149-155.
120. Skalicky AM, Rentz AM, Liu Z, Said Q, Nakagawa JA, Frost MD, et al. Economic burden, work, and school productivity in individuals with tuberous sclerosis and their families. J Med Econ. 2018;21(10):953-959.
121. Skalicky AM, Rentz AM, Liu Z, Wheless JW, Pelletier CL, Dunn DW, et al. The burden of subependymal giant cell astrocytomas associated with tuberous sclerosis complex: Results of a patient and caregiver survey. J Child Neurol. 2015;30(5):563-569.
122. Srivastava S, Prohl AK, Scherrer B, Kapur K, Krueger DA, Warfield SK, et al. Cerebellar volume as an imaging marker of development in infants with tuberous sclerosis complex. Neurology. 2018;90(17):e1493-e1500.
123. Staley BA, Montenegro MA, Major P, Muzykewicz DA, Halpern EF, Kopp CM, et al. Self-injurious behavior and tuberous sclerosis complex: Frequency and possible associations in a population of 257 patients. Epilepsy Behav. 2008;13(4):650-653.
124. Steinhausen H, von Gontard A, Spohr H, Hauffa B, Eiholzer U, Backes M, et al. Behavioral phenotypes in four mental retardation syndromes: Fetal alcohol syndrome,

Prader-Willi syndrome, fragile X syndrome, and tuberous sclerosis. Am J Med Genet. 2002;111(4):381-387.

125. Tierney KM, McCartney DL, Serfontein JR, de Vries PJ. Neuropsychological attention skills and related behaviours in adults with tuberous sclerosis complex. Behav Genet. 2011;41(3):437-444.

126. Toldo I, Brasson V, Miscioscia M, Pelizza MF, Manara R, Sartori S, et al. Tuberous sclerosis-associated neuropsychiatric disorders: A paediatric cohort study. Dev Med Child Neurol. 2019;61(2):168-173.

127. Trickett J, Heald, M, Oliver C, Richards C. A cross-syndrome cohort comparison of sleep disturbance in children with Smith-Magenis syndrome, Angelman syndrome, autism spectrum disorder and tuberous sclerosis complex. J Neurodev Disord. 2018;10(1):9.

128. Tritton T, Bennett B, Brohan E, Grant L, Cooper A, Fladrowski C, et al. Health utilities and quality of life in individuals with tuberous sclerosis complex (TSC) who experience epileptic seizures: A web-based survey. Epilepsy Behav. 2019;92:213-220.

129. Tye C, Farroni T, Volein A, Mercure E, Tucker L, Johnson MH, et al. Autism diagnosis differentiates neurophysiological responses to faces in adults with tuberous sclerosis complex. J Neurodev Disord. 2015;7(1):33.

130. Tye C, Thomas LE, Sampson JR, Lewis J, O'Callaghan F, Yates JR, et al. Secular changes in severity of intellectual disability in tuberous sclerosis complex: A reflection of improved identification and treatment of epileptic spasms? Epilepsia Open. 2018;3(2):276-280.

131. van Eeghen AM, Black ME, Pulsifer MB, Kwiatkowksi DJ, Thiele EA. Genotype and cognitive phenotype of patients with tuberous sclerosis complex. Eur J Hum Genet. 2012;20(5):510-515.

132. van Eeghen AM, Chu-Shore CJ, Pulsifer MB, Camposano SE, Thiele EA. Cognitive and adaptive development of patients with tuberous sclerosis complex: A retrospective, longitudinal investigation. Epilepsy Behav. 2012;23(1):10-15.

133. van Eeghen AM, Numis AI, Staley BA, Therrien SA, Thibert RL, Thiele EA. Characterizing sleep disorders of adults with tuberous sclerosis complex: A questionnaire-based study and review. Epilepsy Behav. 2011;20(1):68-74.

134. Vergeer M, de Ranitz-Greven WL, Neary MP, Ionescu-Ittu R, Emond B, Duh MS, et al. Epilepsy, impaired functioning, and quality of life in patients with tuberous sclerosis complex. Epilepsia Open. 2019;4(4): 581-592.

135. Vignoli A, La Briola F, Peron A, Turner K, Vannicola C, Saccani M, et al. Autism spectrum disorder in tuberous sclerosis: Searching for risk markers. Orphanet J Rare Dis. 2015;10(1):154.
136. Vignoli A, La Briola F, Turner K, Scornavacca G, Chiesa V, Zambrelli E, et al. Epilepsy in TSC: Certain etiology does not mean certain prognosis. Epilepsia. 2013;54(12):2134-2142.
137. Walz NC, Byars AW, Egelhoff JC, Franz DN. Supratentorial tuber location and autism in tuberous sclerosis complex. J Child Neurol. 2002;17(1):830-832.
138. Wang Y, Pang L, Ma S, Zhang M, Liu L, Zou L. Epilepsy may be the major risk factor of mental retardation in children with tuberous sclerosis: A retrospective cohort study. Epilepsy Behav. 2017;77:13-18.
139. Webb DW, Fryer AE, Osborne JP. On the incidence of fits and mental retardation in tuberous sclerosis. J Med Genet. 1991;28(6):395-397.
140. Webb DW, Thomson JL, Osborne JP. Cranial magnetic resonance imaging in patients with tuberous sclerosis and normal intellect. Arch Dis Child. 1991;66(12):1375-1377.
141. Weber AM, Egelhoff JC, McKellop JM, Franz DN. Autism and the cerebellum: Evidence from tuberous sclerosis. J Autism Dev Disord. 2000;30(6):511-517.
142. Whitehead LC, Gosling V. Parent’s perceptions of interactions with health professionals in the pathway to gaining a diagnosis of tuberous sclerosis (TS) and beyond. Res Dev Disabil. 2003;24(2):109-119.
143. Wilbur C, Sanguansermsri C, Chable H, Anghelina M, Peinhof S, Anderson K, et al. Manifestations of tuberous sclerosis complex: The experience of a provincial clinic. Can J Neurol Sci. 2017;44(1):35-43.
144. Wilde L, Eden K, de Vries P, Moss J, Welham A, Oliver C. Self-injury and aggression in adults with tuberous sclerosis complex: Frequency, associated person characteristics, and implications for assessment. Res Dev Disabil. 2017;64:119-130.
145. Wilde L, Wade K, Eden K, Moss J, de Vries PJ, Oliver C. Persistence of self-injury, aggression and property destruction in children and adults with tuberous sclerosis complex. J Intellect Disabil Res. 2018;62(12):1058-1071.
146. Winterkorn EB, Pulsifer MB, Thiele EA. Cognitive prognosis of patients with tuberous sclerosis complex. Neurology. 2007;68(1):62-64.
147. Wong HT, McCartney DL, Lewis JC, Sampson JR, Howe CH, de Vries PJ. Intellectual ability in tuberous sclerosis complex correlates with predicted effects of mutations on TSC1 and TSC2 proteins. J Med Genet. 2015;52:815-822.

148. Wong V. Study of the relationship between tuberous sclerosis complex and autistic disorder. J Child Neurol. 2006;21(3):199-204.
149. Yang G, Shi ZN, Meng Y, Shi XY, Pang LY, Ma SF, et al. Phenotypic and genotypic characterization of Chinese children diagnosed with tuberous sclerosis complex. Clin Genet. 2017;91(5):764-768.
150. Yapici Z, Dörtcan N, Baykan BB, Okan F, Dinçer A, Baykal C, et al. Neurological aspects of tuberous sclerosis in relation to MRI/MR spectroscopy findings in children with epilepsy. Neurol Res. 2007;29(5):449-454.
151. Yu C, Lin F, Zhao L, Ye J, Qin W. Occult white matter damage contributes to intellectual disability in tuberous sclerosis complex. Intelligence. 2009;37(2):174-180.
152. Yum M, Lee EH, Ko T. Vigabatrin and mental retardation in tuberous sclerosis: infantile spasms versus focal seizures. J Child Neurol. 2013;28(3):308-313.
153. Zaroff CM, Barr WB, Carlson C, LaJoie J, Madhavan D, Miles DK, et al. Mental retardation and relation to seizure and tuber burden in tuberous sclerosis complex. Seizure. 2006;15(7):558-562.
