# Supplementary material for: The research landscape of tuberous sclerosis complex–associated neuropsychiatric disorders (TAND)—a comprehensive scoping review
Source: J Neurodev Disord. 2022 Feb 13;14:13. doi: 10.1186/s11689-022-09423-3 (PMC8853020; doi:10.1186/s11689-022-09423-3)
Supplement: Supplementary file 3 — Additional file 3. Quality rating categorisation and grouping. [file 11689_2022_9423_MOESM3_ESM.pdf]

Additional File 3

Step 1: Quality rating categorisation

Both the ARRIVE and MMAT quality appraisal tools for animal studies and human studies respectively were evaluated according to excellent, good and poor categorisations. Each criterion was evaluated according to this three point system. If the criterion was clearly addressed, the study would be rated as excellent and colour coded as green for this aspect. If the criterion was partially addressed within the study, but lacked the level of detail to receive an excellent evaluation, the study would be rated as good and colour coded as orange for this aspect. If the criterion was relevant to the study but not outlined specifically within the main text, the study would be rated as poor and colour coded as red for this aspect. Some criteria were not relevant to the study in question. This was particularly true for MMAT criterion 5 for quantitative descriptive studies (Is the statistical analysis appropriate to answer the research question?), which could not be answered for case studies and cohort studies where statistical analyses were not appropriate. These criterion were deemed not applicable, and were not included when the next stage of quality appraisal 'grouping' was conducted.

Step 2: Quality rating grouping

Once all studies had been evaluated according to relevant criteria for their study design and colour coded as above, the total number of green, orange, and red categorisations were grouped more meaningfully, as supported by the TANDem project statistician (SGL). Animal studies were evaluated according to 38 individual criteria, and therefore grouping was relatively difficult. Animal studies were grouped into two quality distinctions: high and relatively high based on the number of individual poor ratings per study. Studies with fewer than ten 'poor' criteria were rated as high quality, and studies with ten or more 'poor' criteria were rated as relatively high. Case studies were grouped into two quality distinctions: high and relatively high. Grouping decisions are outlined in more detail in Table 1 below. There was greater variability in criteria evaluations that could be assigned to cohort studies, which were best represented according to four quality distinctions: low, adequate, relatively high, and high. Grouping decisions are outlined in more detail in Table 2 below.

Table 1: Case study groupings

High			Relatively High		
4	0	1	3	0	2
5	0	1	3	0	1
			3	1	1
			4	2	1

Table 2: Cohort study groupings

High			Relatively High			Adequate			Low		
5	0	0	6	1	0	3	2	1	2	3	2
6	0	0	3	2	0	4	2	1	2	1	3
6	0	0	4	2	0	3	3	1	3	1	3
			5	2	0	2	4	1			
			3	3	0	5	0	2			
			4	3	0	4	1	2			
			1	4	0	3	2	2			
			3	4	0						
			1	5	0						
			6	0	1						
			5	1	1						
